# Reconcilable differences: Using retrospective photogrammetry to bridge the divide between analogue and digital site data collected during long-term excavation projects

**DOI:** 10.1371/journal.pone.0310741

**Published:** 2024-11-21

**Authors:** Ole Fredrik Unhammer, Magnus Mathisen Haaland, Simon James Armitage, Christopher Stuart Henshilwood, Karen Loise van Niekerk

**Affiliations:** 1 Department of Archaeology, History, Cultural Studies and Religion, University of Bergen, Bergen, Norway; 2 SFF Centre for Early Sapiens Behaviour (SapienCE), University of Bergen, Bergen, Norway; 3 Norwegian Institute for Cultural Heritage Research, Oslo, Norway; 4 University of Stavanger—Museum of Archaeology, Stavanger, Norway; 5 Department of Geography, Centre for Quaternary Research, Royal Holloway University of London, Egham, Surrey, United Kingdom; 6 Evolutionary Studies Institute, University of the Witwatersrand, Johannesburg, South Africa; New York University, UNITED STATES OF AMERICA

## Abstract

Over the last 30 years, high-resolution site documentation has rapidly developed, with analogue drawings and film photography being replaced with high-precision digital recordings. Today, most archaeological field data sets are produced using digital tools that store spatial and visual information in various digital formats directly, i.e., *born-digital*. A fully digital workflow makes the process of combining, comparing, and integrating field datasets quicker, easier, and potentially more analytically powerful. However, at sites where both analogue and born-digital data sets have been produced, additional procedural digitization steps are required before full data interoperability is achieved. In cases where the archaeological sites have a long excavation history, multiple generations of analogue and digital site documentation techniques have often been used, making it particularly challenging to physically reconstruct an excavated site based on its archival material. The Middle Stone Age site of Blombos Cave, South Africa, is a prime example of this type of challenging situation. This site features a more than 3-meter-deep and well-preserved archaeological sequence dated to between 300 and 100 000 years ago. Since it was initially excavated in 1991, multiple archaeological campaigns have been carried out (>15), and the excavations are still ongoing. The field documentation from Blombos Cave has, over the years, produced varied but rich datasets that have never been integrated into a single, coherent, and accessible archive. In this paper we evaluate the changes in excavation protocol at Blombos Cave over time, and we use this knowledge to digitally integrate and map the various stages of excavation within a three-dimensional framework using digital photogrammetry and archival photographs. The archaeological and analytical value of this approach is exemplified through multiple case studies, in which we demonstrate how and why the merging of old and new archaeological field data can lead to new results, specifically by offering more complete mapping and more accurate and analytically dynamic visualisations. The research history at Blombos Cave is not unique or site-specific. Our approach would be applicable to a wide variety of sites and contexts where long-running excavations have produced a mix of analogue and digital field data.

## Introduction

According to Carver [1: 123] an archaeological deposit is a “three-dimensional artefact, only seen once [during excavation], and never seen whole”. In other words, once a site has been excavated, usually in reverse chronological order of its formation, it cannot be put back together again in the same physical state in which it was initially dug. As such, an archaeological excavation represents a type of destructive scientific experiment that is hard to evaluate independently, because it cannot be replicated after the process of observation (excavation) has occurred. The non-reproducible nature of archaeological excavations–combined with a growing awareness of the importance of depositional context and artefact provenience–has over time driven archaeologists to adopt gradually more advanced documentation strategies [2–5]. While many of these strategies involve the combined recording of the visual, spatial, physical and contextual aspects of a site at various stages of excavation, no formal or universally acknowledged documentation procedure in field archaeology has been adopted beyond that of loosely defined best-practice guidelines [1,6].

Digital photogrammetry is a documentation method that in the last decade has become widely used within the field of archaeology at multiple spatial levels, including landscape, site, and artefact recording [7–10]. Widespread adoption of the technique is due to its precision, low cost, and ease of application in most cases [11–16]. As an easily accessible field method, it has become one of the most common ways to document the spatial configuration of an archaeological site at different stages of excavation, enabling the production of 3D snapshot models that later can be digitally combined and viewed as a whole [17–21]. The method itself uses digital still images taken of an object from different angles to calculate and reconstruct the surface of that object in three dimensions (3D). When capturing these images, emphasis should be put on achieving the sharpest images possible with minimal distortion. Important values for camera settings are therefore a low ISO value, optimum aperture, and high shutter speed to avoid motion blur. A fixed lens with a focal length that causes minimal distortion (35 mm film equivalent) is generally recommended although higher and lower focal length will work. Capturing with a higher resolution camera will allow for more detail captured in each image and recoding in a lossless format will ensure that this information is preserved optimally. The resultant 3D model can be precisely scaled, oriented and geographically positioned within a site coordinate system using reference locations known as Ground Control Points (GCP) [22].

Typically, modern photogrammetric work is conducted using images specifically obtained for the purpose, but in principle archive images taken for other purposes may also be used. This practice dates to the invention of the method 100 years ago [23–25]. The digital reconstruction of archaeological sites based on archive images has been demonstrated on multiple occasions but has mostly focused on destroyed buildings, structures and features [26–30]. Fewer studies have used archive images to reconstruct archaeological excavations. These have explored the potential of the technique by reconstructing quadrant surfaces [31] and creating models of smaller areas [21] up to whole sites [32–35] to monitor site degradation and plan for rehabilitation. The technique has also been used to orient and link a new investigation into a site that was excavated in the past [36]. A term suggested for the use of archive photographs in photogrammetric modelling with the purpose of obtaining new information and facilitating new investigations is *retrospective photogrammetry* [32]. A frequently reported challenge in several of these studies is the low number of useable archive images and lack of information regarding camera configuration (camera body and lens) and settings (focal length, shutter speed, iso etc.) used during recording and metadata such as time of capture (in the case of analogue photographs). Both issues can lead to either inaccuracies within the resulting model or the inability to produce a viable model. The former problem can occasionally be solved if the studied features are still present at the site and supplementary photographs can be captured [32] whereas the latter may be resolved if the original camera model and setup can be determined [37].

In cases where archaeological sites have a long excavation history, multiple generations of analogue and digital site documentation techniques have often been used, making it particularly challenging to reconstruct the various steps of the excavation site based on the field records in various formats, qualities, and conditions. The Middle Stone Age (MSA) site of Blombos Cave (BBC), South Africa, is a prime example of this type of challenging situation. Blombos Cave features a >3 m deep, well-preserved archaeological sequence dated to between 300 and 100 000 years ago. Since it was initially excavated in 1991, multiple archaeological campaigns have been carried out (>15), and the excavations are still ongoing. The photographic field documentation from BBC has, over the years, recorded the gradual removal of the sediments, but the resulting photographs were not systematically integrated into a single, coherent, and accessible archive. A change in field documentation practices over time is not unique to BBC and is indeed characteristic for many long-term research projects. However, in common with other sites, the evolution of recording methods over time has prevented site-wide data integration and an overall site evaluation.

In this paper we evaluate the changes in excavation protocol at BBC over time and use this knowledge to digitally integrate and map the various stages of excavation within a three-dimensional framework using digital photogrammetry and archival photographs. Photogrammetry has previously been used to map the interior and exterior surfaces of BBC in 3D [38] and to link microstratigraphic observations with the surrounding archaeological context [39–41]. By significantly expanding the extent of image-based site reconstruction already conducted at the site, a more systematic, site-wide investigation of the main trenches and the archaeological record within them is possible.

## Site background

**Fig 1:**
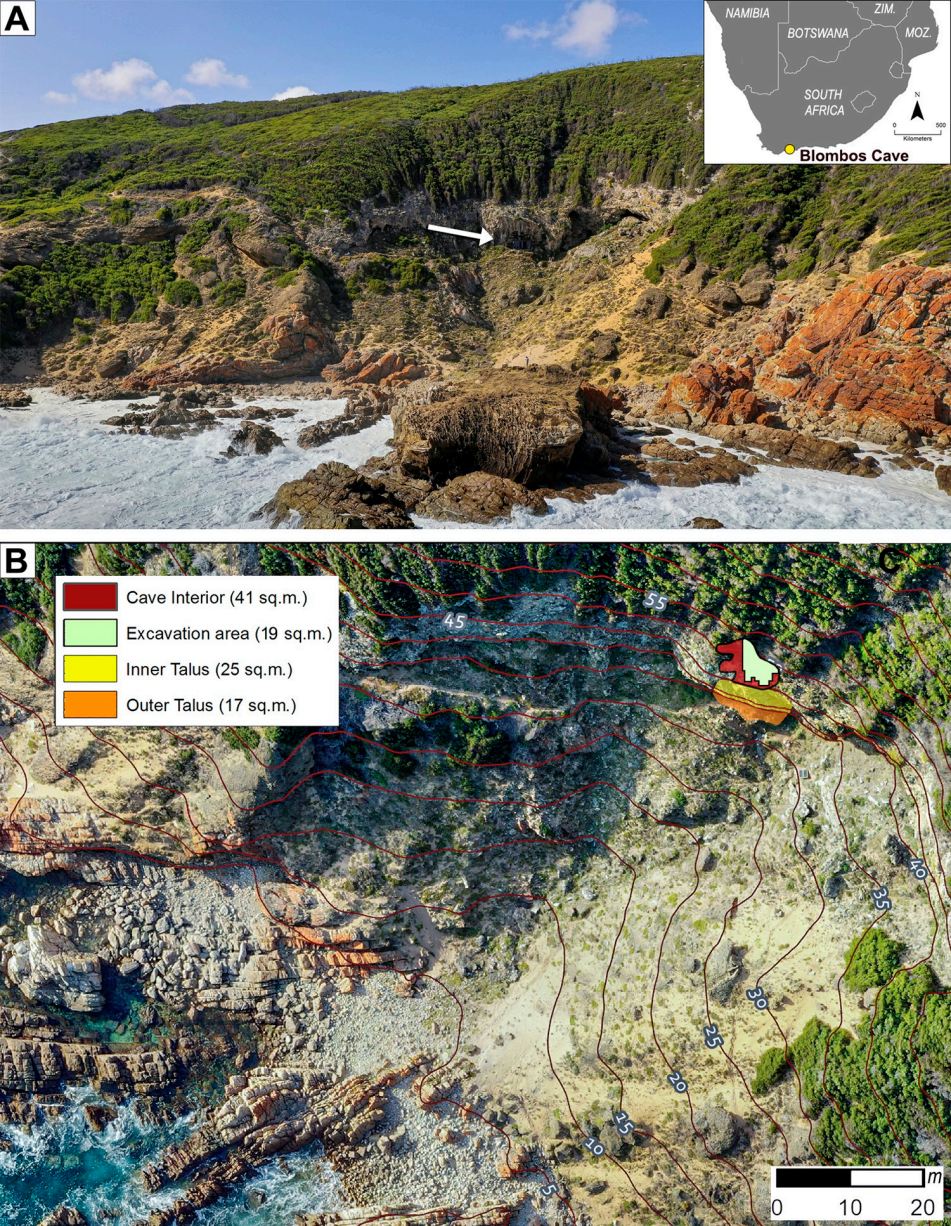
The location of Blombos Cave. (A) Cave location viewed from seaward, and (inset) on a map of southern Africa. (B) Plan view of the embayment containing Blombos Cave, with the extent of cave and talus deposits. A little under half of the cave interior has been excavated (in 2020) while most of the exterior talus remains untouched.

**Fig 2:**
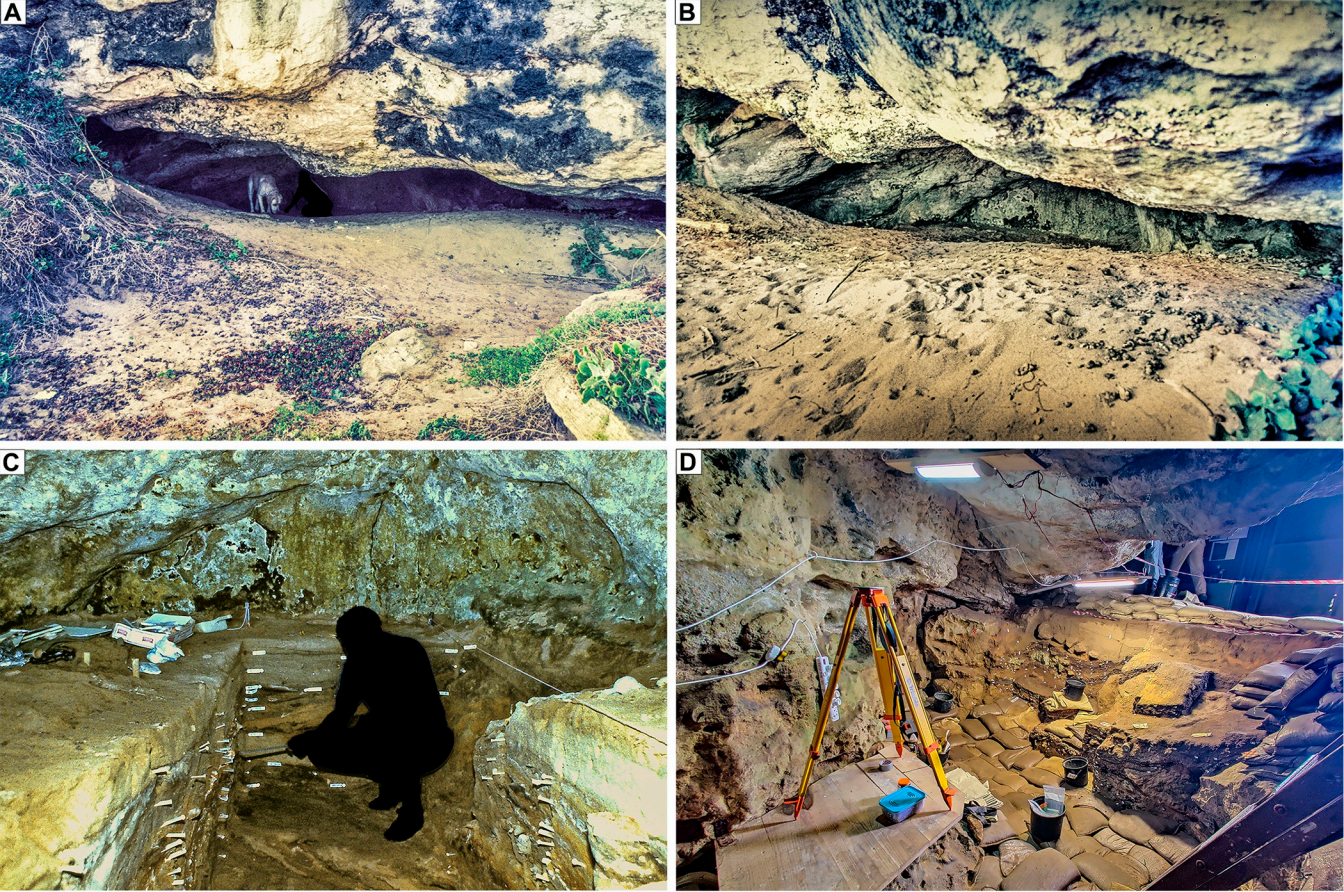
Blombos Cave at different stages of excavation. (A, B) Prior to initial excavation in 1991, (C) the extent of excavations in 1998 and (D) 2020.

**Fig 3:**
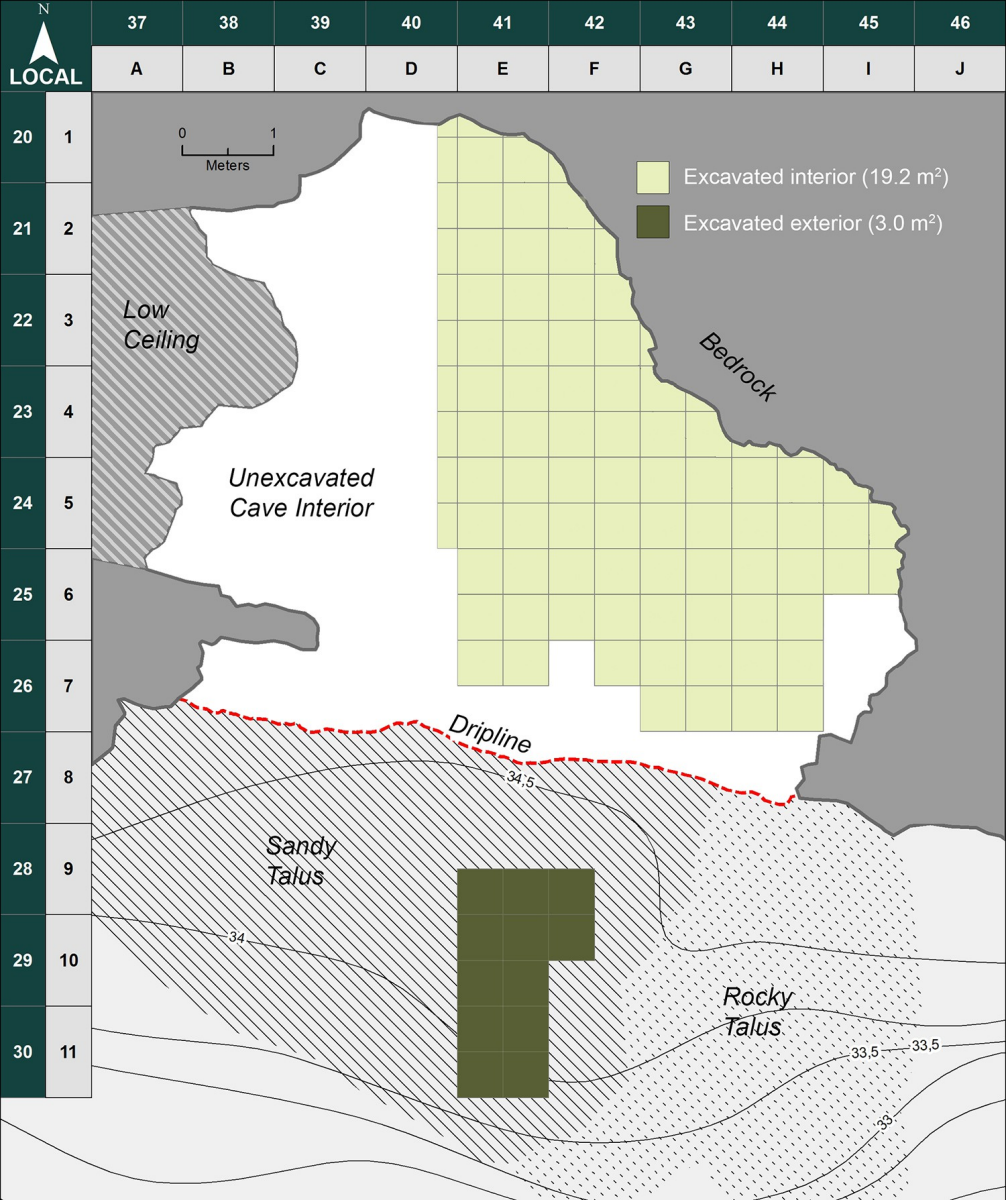
Plan of Blombos Cave. Excavation layout showing the partly or completely excavated areas of the interior (19.2 m2 by 2020) and exterior talus (3 m2 trench, excavated and infilled in 1999).

**Fig 4:**
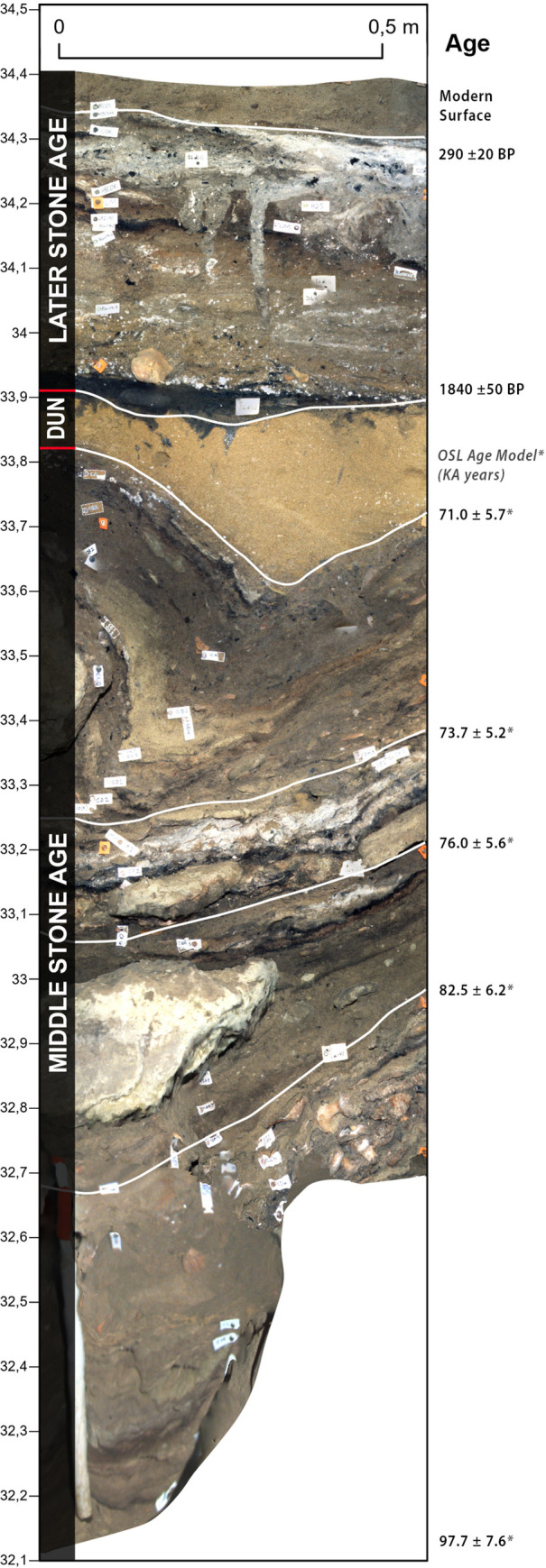
Blombos Cave site stratigraphy. The Later Stone Age (LSA) and Middle Stone Age (MSA) stratigraphic sequence at Blombos Cave as seen at the centre of the cave from a westward facing perspective of section E4c west (41x, -24 - -23.5y).

Blombos Cave is located ~ 300 km east of Cape Town on the southern coast of South Africa (34° 25’S, 21°13’E). The cave is situated in a south-facing calcarenite cliff, ~34.5 m above modern sea level and ~100 m from the present-day shoreline (Fig 1). It was first surveyed in 1990, when the entrance of the cave was found to be partially sealed by recent dune sand (Fig 2A and 2B). Between 1991 and 2020, eighteen field seasons have been carried out during which an interior area of ~19.2 m^2^, and a talus area of ~3 m^2^ have been excavated down to 2–3 meters depth (Figs 2C, 2D and 3). The excavated archaeological sequence consists of a Later Stone Age (LSA) component near the surface, dated to 2000–290 cal. year BP [42], underlain by a thicker MSA component ranging in age from 97.7 ± 7.6 and 71.0 ± 5.7 ka [43] (Fig 4).

The archaeological assemblage recovered from MSA layers at BBC have provided empirical, chronological, ecological, and contextual evidence for when and where key behavioural innovations first emerged amongst anatomically modern *Homo sapiens* populations in southern Africa. These innovations include the early adoption of multi-step bone and stone tool technology [44–46], the manufacture and use of personal ornaments [47–49], evidence of ochre paint production [50], as well as the earliest known occurrence of geometric patterns on portable objects [51,52].

### Three decades of archaeological excavations: A review of the Blombos Cave documentation systems

**Fig 5:**
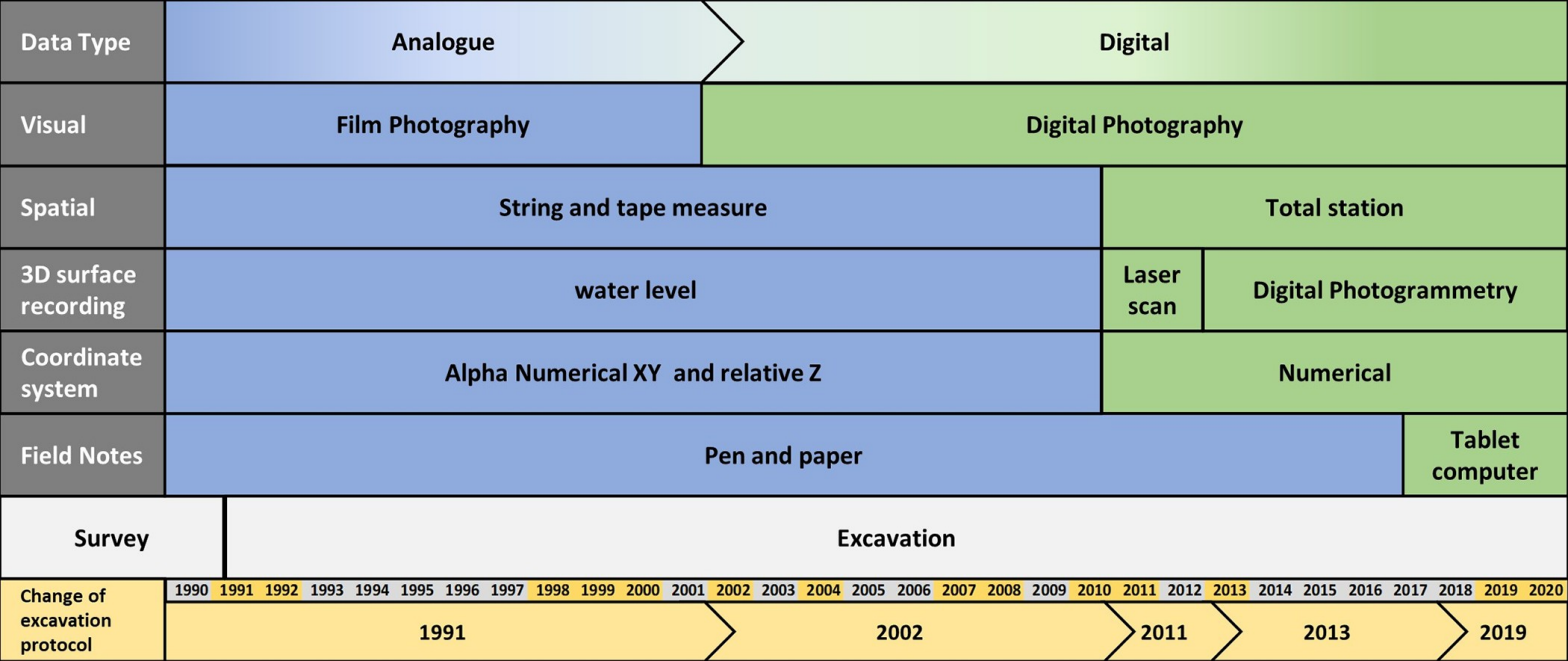
The evolution of the documentation methodology at Blombos Cave between 1991 and 2020. Initial excavations (1991) adopted a fully analogue system with a gradual transition towards a fully digital system by 2019.

The archaeological investigation of BBC can be divided into two main periods. In 1990 the site was surveyed and in 1991–1992 the first stage of excavation took place, as part of a larger study of LSA sites located within the Blombosfontein Nature reserve [53]. The second, ongoing, stage of investigation began in 1997, focusing on the MSA deposits. Over the last three decades, changes in research priorities combined with the development of cheaper, more accessible, and more comprehensive digital recording techniques have led to significant changes and improvements of the original field documentation protocol that was established in the early 1990s. Fig 5 provides a graphical overview of when key aspects of the BBC field recording procedures changed. This overview has allowed us to identify five *phases* of documentation practice; the first phase represents the establishment of the initial documentation system, while the four subsequent phases are characterised by the stepwise abandonment of analogue documentation methods in favour of digital recording techniques.

The documentation record which acts as the material for this paper was collected during archaeological excavations conducted under the following permits issued to CSH: 8/96/06/001/51 (1998–1999), 80/00/01/006/51 (2000 and 2002), 2003/12-001 (2004), 2005/05-005 (2005), 2007/03-003 (2007–2009), 2010/02-001 (2010), 2011/09/001 (2011 and 2013), 18101602AS1017F (2019 and 2020). Permits were issued by the National Monuments Counsel (1998 and 1999) and Heritage Western Cape (2000–2020) under the National Heritage Resource Act (Act 25 of 1999) and the Western Cape Provincial Gazette 6061, Notice 298 of 2003. A copy of the material is stored and curated at the University of the Witwatersrand Satellite Laboratory, Buitenkant Street, Cape Town. All necessary permits were obtained for the described study, which complied with all relevant regulations.

During the first phases of documentation (1991 to 2001) the 3D location of features, artefacts and surfaces was recorded using an analogue water-level device in combination with a hand-held tape measure [54–56]. Each measurement was recorded in relation to the local grid within each excavation quadrat (50x50 cm), which in turn was part of an alphanumerical grid that covered the entire site and acts as the sites local coordinate system. All visual documentation was conducted using analogue film photography and field notes/drawings were handwritten [53]. During the second documentation phase (2002–2010), the introduction of digital cameras led to a significant increase in visual documentation, though the analogue methods for recording location were retained [50,57,58].

The third documentation phase (2011–2013) is characterized by the implementation of total station measurements, replacing the analogue system using tape measure and water level. With the introduction of the total station a site wide numeric grid location system was instigated, though physical excavation still follows the alphanumeric grid squares naming convention. The new numerical coordinate system was aligned with the alphanumerical predecessor (Fig 3) ensuring a consistent local excavation layout. All spatial measurements from this point onwards were conducted within a single site-wide coordinate system as opposed to the previous method which kept each analogue measurement locally constrained within individual quadrants. The total station increased both the accuracy and precision of spatial measurements, and in combination with a 3D terrestrial laser scanner, the exact topography of the cave’s interior and exterior was digitally documented for the first time [59].

The fourth documentation phase (2013–2018) is characterized by introduction of digital photogrammetry [38]. Photogrammetry was used to create high-resolution 3D reconstructions of the interior and exterior of the site. Analysis of the photogrammetric results suggests that their accuracy is comparable to those produced by a laser scanner, though digital photogrammetry is cheaper, more flexible and can provide photorealistic textures and orthophotographs in addition to 3D point clouds. Because of these advantages, photogrammetry has been the primary tool for both visual and spatial surface recording at BBC since 2013.

The fifth (current) documentation phase in BBC (2019 onwards) is characterized by the adoption of a fully digital documentation workflow in which all contextual information is systematically recorded on portable computer tablets and organized within a single database system [39].

### Evaluating the Blombos Cave site records: Towards a site-wide integration of digital and analogue data using archive photographs

Our review of the BBC documentation systems demonstrates that the implementation of digital documentation methods has not been a linear process but can best be characterized as an opportunistic, curiosity-driven, and exploratory process in which new recording techniques gradually became integrated into the excavation protocol. Consequently, the format and nature of data collection has been multifaceted and mosaic, which needs to be considered when different types of field data are compiled and organized within a single system.

Photographs of BBC excavations are the most consistently collected type of field data. During the first 10 years of field work, all photographs were captured on film, and the level of detail captured in these analogue images often exceeds that of the first-generation digital photographs. Prior to 2021, the BBC photographic archive consisted of 22,438 photographs, of which more than half document section walls, surfaces and features. The size, content, and quality of the Blombos Cave photographic archive makes it an ideal dataset for testing the use of retrospective photogrammetry to reconstruct stages of excavation. Our general workflow–which involves the combined use of photogrammetric procedures, archive photographs and GIS software—consists of five main procedural steps:

1. Digitizing (where necessary) and categorising the BBC photographic archive.
2. Reviewing and selecting photographic datasets that are conducive to retrospective photogrammetry.
3. Producing 3D models of the site at different stages of excavation.
4. Georeferencing the 3D models and evaluating their accuracy.
5. Analytically activating the retrospective 3D models by using them to conduct large-scale, cross-seasonal evaluations

The archaeological and analytical value of our approach is exemplified through multiple case studies, in which we demonstrate how and why the merging of analogue and digital field documentation can lead to entirely new and unprecedented results, including:

- A site-wide assessment of the cave’s overall topography, morphology, and excavated trenches.
- A complete evaluation of the stratigraphic relationships between major occupational periods (i.e., the MSA and the LSA) and between different MSA occupation phases.
- The mapping of single-context occupation surfaces excavated over multiple seasons.
- The visualisation and spatial analysis of MSA artefacts recorded over multiple seasons.

## Methods

### Digitalising and organising the Blombos Cave photographic archive

At the beginning of this study, the BBC photographic archive consisted of 35 mm film slides captured between 1990–2000 (n = 1400 analogue photos) and digital photographs captured with various digital cameras between 2002–2020 (n = 21038 digital photos). Slides were digitalised using a Nikon Super Coolscan 8000 ED slide scanner yielding digital images with a resolution of 4000 DPI (5905X4032 pixels). During this process contextual information handwritten on the slides was also digitally transcribed. Once the entire BBC photographic dataset (n = 22438) had been digitized, they were systematically organized within a simple folder system, first by year and date, and then by location, subject and archaeological context. An overview of the photographic equipment and cameras used, and the approximate number of images taken per excavation season is provided in Table 1.

**Table 1:** Recording format, equipment and image archive size per excavation season.

Year	Original format	Camera model	Lens	Photos captured
**1998–2000**	Analogue	Digitalized with a Nikon Super Coolscan 8000	N/A	Ca. 1400
**2002**	Digital	Nikon Coolpix E995	Nikkor 8-32mm f/2.6–5.1	433
**2004**		Nikon D70, Sony DSC-U60	Nikon 18-70mm f/3.5–4.5 AF-S DX, 5-33mm f/2.8	943
**2005**		Nikon D70 Nikon Coolpix E5900	Nikon 18-70mm f/3.5–4.5 AF-S DX, 7.8–23.4mm f/2.8–4.9	508
**2006**		Canon PowerShot S80	5.8–20.7mm f/2.8–5.3	73
**2007**		Nikon Coolpix E5900 Canon PowerShot S80	7.8–23.4mm f/2.8–4.9 5.8–20.7mm f/2.8–5.3	606
**2008**		Nikon Coolpix E5900 Canon PowerShot S80 Canon EOS 400D	7.8–23.4mm f/2.8–4.9 5.8–20.7mm f/2.8–5.3 Canon EF-S 18-55mm f/3.5–5.6	838
**2009**		Nikon D70	Nikon 18-70mm f/3.5–4.5 AF-S DX	405
**2010**		Nikon D70	Nikon 18-70mm f/3.5–4.5 AF-S DX	700
**2011**		Nikon D700	Nikon Nikkor AF-S 24-70/2,8 E ED VR	Ca. 1300
**2013**		Nikon D4	Nikon Nikkor AF-S 24-70/2,8 E ED VR	Ca. 2200
**2018**		Nikon D4	Nikon Nikkor AF-S 24-70/2,8 E ED VR	74
**2019**		Nikon D4, DJI Mavic Pro II (Hasselblad L1D-20c), Sony DSC-RX100M5	Nikon Nikkor AF-S 24-70/2,8 E ED VR Hasselblad L1D-20c 28mm f2.2 Zeiss 8.8–25.7mm f/1.8–2.8	12958

Archive photographs taken during excavation seasons at Blombos cave from 1991–2020. The duration of each field season has varied and the season from which some analogue images came from could not be determined. n/a indicates data not available.

### Reviewing and selecting photographic datasets conducive to retrospective photogrammetry

**Fig 6:**
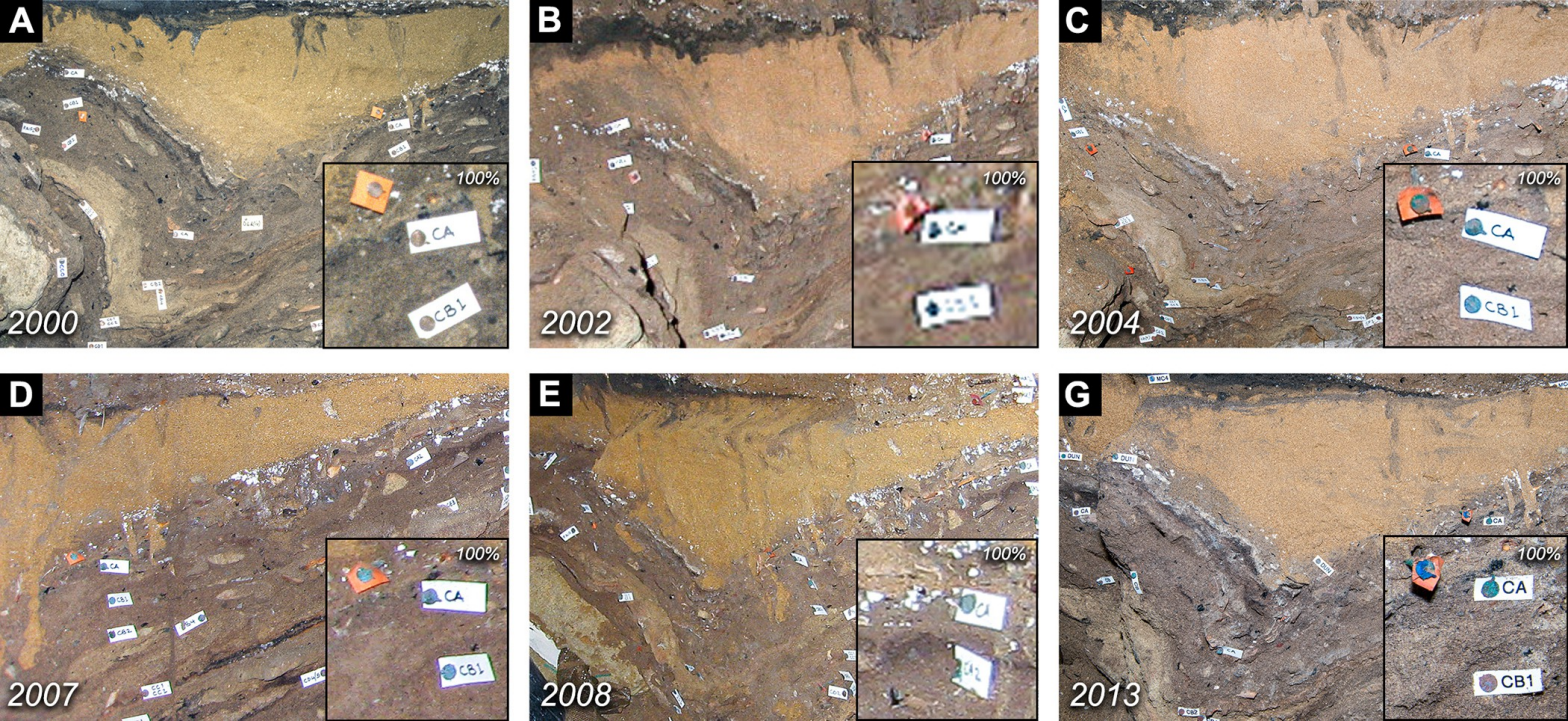
The image quality varies greatly. Resolution, colour, depth sharpness and available metadata varies greatly across the archive. This is illustrated with images from the western profile. (A) (analogue) is significantly sharper and more detailed than (BDE) the early digital images. Changes in lighting and colour balance also affects the visible information e.g. A and D have a very flat light while B, C and G have sharper illumination and a greater angle between the light source and the camera, creating small shadows.

A systematic review of the re-organized BBC image archive was carried out on a season-by-season basis with the purpose of identifying photographs particularly suited for reconstructing section walls, surfaces, and general cave topography. This process revealed that the resolution, image quality (colour depth, exposure, noise, motion blur etc.), camera parameters (focal length, shutter speed, ISO, white balance) and colour temperature varied greatly across seasons due to the different camera models, lenses, camera settings and the original purpose of the image (the recording of a profile, artefact, large scene, excavation members, visitors etc) (Fig 6). For example, the overall quality of the digitalized analogue slides (Fig 6A) was noticeably higher than that for images captured using the first digital cameras (Fig 6B) in part due to the high-quality scanner that was used. However, unlike photographs taken with a digital camera, the digitalized slides do not provide any meta-data revealing the original camera settings or lens parameters. When processing digital slides in our photogrammetric software (Agisoft Metashape Professional) the lack of EXIF-data (Exchangeable image file format) sometimes prevents successful model generation or causes a warping of the model. The variety in colour (both temperature and white balance) is due to colour correction not having been part of the recording strategy at BBC. The result is that many factors including the different camera configurations, white balance settings, changing light sources, different reflective material in the surroundings etc. has dictated the colour reproduction within the archive images. The absence of white balance and colour calibration targets within the archive images prevented us from confidently adjusting the colours. As colours could not be reliable adjusted to achieve a correct representation this was decided against.

Our review of the photographic archive indicated that some section walls could not be reconstructed. The primary reason for the inability to reconstruct certain profile sections was the lack of a sufficient number of overlapping images. A secondary reason was the low quality (resolution, distortion, noise) of the images which prevented successful section reconstruction. The challenges of working with the photographic archive is further elaborated on in the discussion. An overview of the image sets selected for retrospective photogrammetric processing is provided in Table 2.

**Table 2:** Image sets used for retrospective photogrammetry.

Image Sett	Model ID	Year	Model Type	Image count	Camera model	Original Format	ISO	Image format	Aperture	Focal Lenth	Image resolution
**1**	PGA_001	2007–2008	Section Wall	99	Nikon D70, Canon EOS 400D DIGITAL Canon PowerShot S80	Digital	200–1400 400 200–100	.tif .jpg .jpg	F/3.5–6 F/4 F/2.8–3.2	18–34 18–28 5.8–7.3	1488x2240 2000x3008, 2592x3888, 1600x1200 2592x1944 3264x2448
**2**	PGA_002	2011	Section Wall	358	Nikon D700	Digital	200–8000	.tif and.jpg	F/4.5–11	18–70	4256x2832
**3**	PGA_003	2011	Section Wall	40	Nikon D700	Digital	200–3200	.jpg	F/7.1–11	24–55	4256x2832
**4**	PGA_004	2011	Section Wall	16	Nikon D700	Digital	400–2000	.jpg	10–11	32–40	4256x2832
**5**	PGA_005	2011	Section Wall	253	Nikon D700	Digital	200–500	.jpg	9–16	52–70	4256x2832
**6**	PGA_006	2013	Section Wall	26	Nikon D4	Digital	320–400	.jpg	7.1	28–36	4928x3280
**7**	PGA_007	2000	Section Wall	125	Nikon Super Coolscan 8000 ED	Analogue	N/A	.dng	N/A	N/A	5905x4032
**8**	PGA_008	2005	Section Wall	99	Nikon D70, Canon EOS 400D DIGITAL Canon PowerShot S80	Digital	100–1600	.tif .jpg .jpg	3.5–5.3 4 2.8–3.2	18–31 18–28 5.8–7.2	1488x2240 2000x3008, 2592x3888, 1600x1200 2592x1944 3264x2448
**13**	PGA_013	2007	Section Wall	100	Canon PowerShot S80 Nikon Coolpix E5900	Digital	N/A	.jpg .jpg	2.8–4.5	5.8–7.2	2448x3264 2592x1944, 2048x1536 2592x1944
**14**	PGA_014A	2019	Section Wall	463	Nikon D4	Digital	200	.dng	9	35	4928x3280
	PGA_014B	2019	Section Wall	463	Nikon D4	Digital	200	.dng	9	35	4928x3280
	PGA_014C	2019	Section Wall	463	Nikon D4	Digital	200	.dng	9	35	4928x3280
	PGA_014D	2019	Section Wall	463	Nikon D4	Digital	200	.dng	9	35	4928x3280
	PGA_014E	2019	Section Wall	463	Nikon D4	Digital	200	.dng	9	35	4928x3280
	PGA_014F	2019	Section Wall	463	Nikon D4	Digital	200	.dng	9	35	4928x3280
**15**	PGA_015	2019	Section Wall	261	Nikon D4	Digital	200	.dng	9	24–35	4928x3280
**16**	PGA_016A	2019	Section Wall	376	Nikon D4	Digital	200–500	.dng	7.1–10	60	4928x3280
	PGA_016B	2019	Section Wall	376	Nikon D4	Digital	200–500	.dng	7.1–10	60	4928x3280
	PGA_016C	2019	Section Wall	376	Nikon D4	Digital	200–500	.dng	7.1–10	60	4928x3280
	PGA_016D	2019	Section Wall	376	Nikon D4	Digital	200–500	.dng	7.1–10	60	4928x3280
**17**	PGA_017	2013	Section Wall	26	Nikon D4	Digital	320–400	.jpg	7.1	28–32	4928x3280
**19**	PGA_019	2019	Section Wall	165	Nikon D4	Digital	200	.dng	9	35	4928x3280
**20**	PGA_020	2013	Section Wall	37	Nikon D4	Digital	200–4000	.dng	7.1	34–36	4928x3280
**21**	PGA_021	2005	Section Wall	5	Nikon D70	Digital	200	.jpg	3.5–3.8	18–34	2240x1488
**22**	PGA_022A	2010	Section Wall	21	Nikon D70	Digital	N/A	.jpg	5.5–7.1	18–27	3008x2000
	PGA_022B	2010	Section Wall	21	Nikon D70	Digital	N/A	.jpg	5.5–7.1	18–27	3008x2000
**24**	PGA_024	2002	Section Wall	25	Nikon F70, Sony DSC-U60 Nikon E995	Digital	200 160 100	.tif .jpg .tif	3.5 2.8 2.6–3.1	28–35 5 8.2–12.4	1504x1000, 1632x1224, 1024x768
**30**	PGA_030	2019	Section Wall	376	Nikon D4	Digital	200–500	.dng	7.1–10	60	4928x3280
31	PGA_31A	1998–1999	Section Wall	124	Nikon Super Coolscan 8000 ED	Analogue	N/A	.tif	N/A	N/A	5905x4032
	PGA_31B	1998–1999	Section Wall	124	Nikon Super Coolscan 8000 ED	Analogue	N/A	.tif	N/A	N/A	5905x4032
	PGA_31C	1998–1999	Section Wall	124	Nikon Super Coolscan 8000 ED	Analogue	N/A	.tif	N/A	N/A	5905x4032
	PGA_31D	1998–1999	Section Wall	124	Nikon Super Coolscan 8000 ED	Analogue	N/A	.tif	N/A	N/A	5905x4032
	PGA_31E	1998–1999	Section Wall	161	Nikon Super Coolscan 8000 ED	Analogue	N/A	.tif	N/A	N/A	5905x4032
	PGA_31F	1998–1999	Section Wall	161	Nikon Super Coolscan 8000 ED	Analogue	N/A	.tif	N/A	N/A	5905x4032
	PGA_31G	1998–1999	Section Wall	124	Nikon Super Coolscan 8000 ED	Analogue	N/A	.tif	N/A	N/A	5905x4032
	PGA_31H	1998–1999	Section Wall	124	Nikon Super Coolscan 8000 ED	Analogue	N/A	.tif	N/A	N/A	5905x4032
	PGA_31I	1998–1999	Section Wall	124	Nikon Super Coolscan 8000 ED	Analogue	N/A	.tif	N/A	N/A	5905x4032
	PGA_31J	1998–1999	Section Wall	124	Nikon Super Coolscan 8000 ED	Analogue	N/A	.tif	N/A	N/A	5905x4032
**32**	PGA_32A	1999	Section Wall	90	Nikon Super Coolscan 8000 ED	Analogue	N/A	.tif	N/A	N/A	5905x4032
	PGA_32B	1999	Section Wall	90	Nikon Super Coolscan 8000 ED	Analogue	N/A	.tif	N/A	N/A	5905x4032
	PGA_32C	1999	Section Wall	90	Nikon Super Coolscan 8000 ED	Analogue	N/A	.tif	N/A	N/A	5905x4032
	PGA_32D	1999	Section Wall	90	Nikon Super Coolscan 8000 ED	Analogue	N/A	.tif	N/A	N/A	5905x4032
**33**	PGA_33A	1998–2000	Section Wall	175	Nikon Super Coolscan 8000 ED	Analogue	N/A	.tif	N/A	N/A	5905x4032
	PGA_33B	1998–2000	Section Wall	175	Nikon Super Coolscan 8000 ED	Analogue	N/A	.tif	N/A	N/A	5905x4032
**34**	PGA_34A	2000	Section Wall	191	Nikon Super Coolscan 8000 ED	Analogue	N/A	.tif	N/A	N/A	5905x4032
	PGA_34B	2000	Section Wall	191	Nikon Super Coolscan 8000 ED	Analogue	N/A	.tif	N/A	N/A	5905x4032
	PGA_34C	2000	Section Wall	191	Nikon Super Coolscan 8000 ED	Analogue	N/A	.tif	N/A	N/A	5905x4032
	PGA_34D	2000	Section Wall	191	Nikon Super Coolscan 8000 ED	Analogue	N/A	.tif	N/A	N/A	5905x4032
	PGA_34E	2000	Section Wall	191	Nikon Super Coolscan 8000 ED	Analogue	N/A	.tif	N/A	N/A	5905x4032
	PGA_34F	2000	Section Wall	191	Nikon Super Coolscan 8000 ED	Analogue	N/A	.tif	N/A	N/A	5905x4032
**35**	PGA_35	2019	Section Wall	840	Nikon D4	Digital	200–500	.dng	7.1–10	35–60	4928x3280
**36**	PGA_36	2011	Excavation Surface	9	Nikon D700	Digital	400	.jpg	9	24	4256x2832
**37**	PGA_37	2010	Excavation Surface	12	Nikon D70	Digital	N/A	.jpg	4.5–7.1	18	3008x2000
**38**	PGA_38	2011	Excavation Surface	18	Nikon D700	Digital	200–2000	.jpg	10–14	24–70	4256x2832
**39**	PGA_39	2010	Excavation Surface	8	Nikon D70	Digital	N/A	.jpg	3.5–7.1	18	3008x2000
**40**	PGA_40	2011	Excavation Surface	82	Nikon D700	Digital	200–2000	.tif	7.1–13	24–70	4256x2832
**41**	PGA_41	2011	Excavation Surface	185	Nikon D700	Digital	200–1000	.tif	8–11	24–70	4256x2832
**42**	PGA_42	2011	Excavation Surface	140	Nikon D700	Digital	200–3200	.tif	4.5–9	24–70	4256x2832
**43**	PGA_43	2013	Site Model	1148	Nikon D4	Digital	200–800	.jpg and.tif	5.6–14	24–70	4928x3280

For this paper 43 different sets of images were collected and used to generate models. Some of these models where in turn split into different parts, either because the image set contained some alterations to the subject caused by continued excavation, or as a means of separating different sections.

### Producing 3D models of the site at different stages of excavation

The selected archive image sets (**Table 2**) were processed in Agisoft Metashape, following established photogrammetric protocol commonly used in archaeological projects [11]. Because many of the archive images had not been captured with image-based 3D reconstruction in mind, additional procedural steps and procedural workarounds were carried out to ensure robust results. These problems and their solutions are discussed in the section entitled: What practical challenges were encountered when working with the BBC archive material and how did we solve them?.

### Georeferencing the 3D models and evaluating their accuracy

Many of the 3D models made from photographs captured after 2011 were directly georeferenced using ground control points (GCPs) surveyed by a total station with a nominal uncertainty of ±2 mm + 2 ppm [60]. 3D models made from photographs captured before 2011, when a total station was not used, could not be georeferenced in this way. Instead, these models were indirectly georeferenced, i.e., the GCPs were not independently surveyed in the field but were selected from spatially overlapping post-2011 models. In practice, we used GCPs with three levels of accuracy to create 3D models with respective accuracy levels; (1) Primary GCPs, measured directly with a total station, were used to create directly referenced models (high accuracy); (2) Secondary GCPs extracted from a directly referenced 3D model were used to create indirectly referenced models (medium accuracy); and (3) Tertiary GCPs extracted from indirectly referenced 3D models were in turn used to create a subsequent group of 3D models (low accuracy).

### Using the retrospective 3D models for cross-seasonal site-wide analysis

#### Assessment of the cave’s overall topography, morphology, and excavated trenches

**Fig 7:**
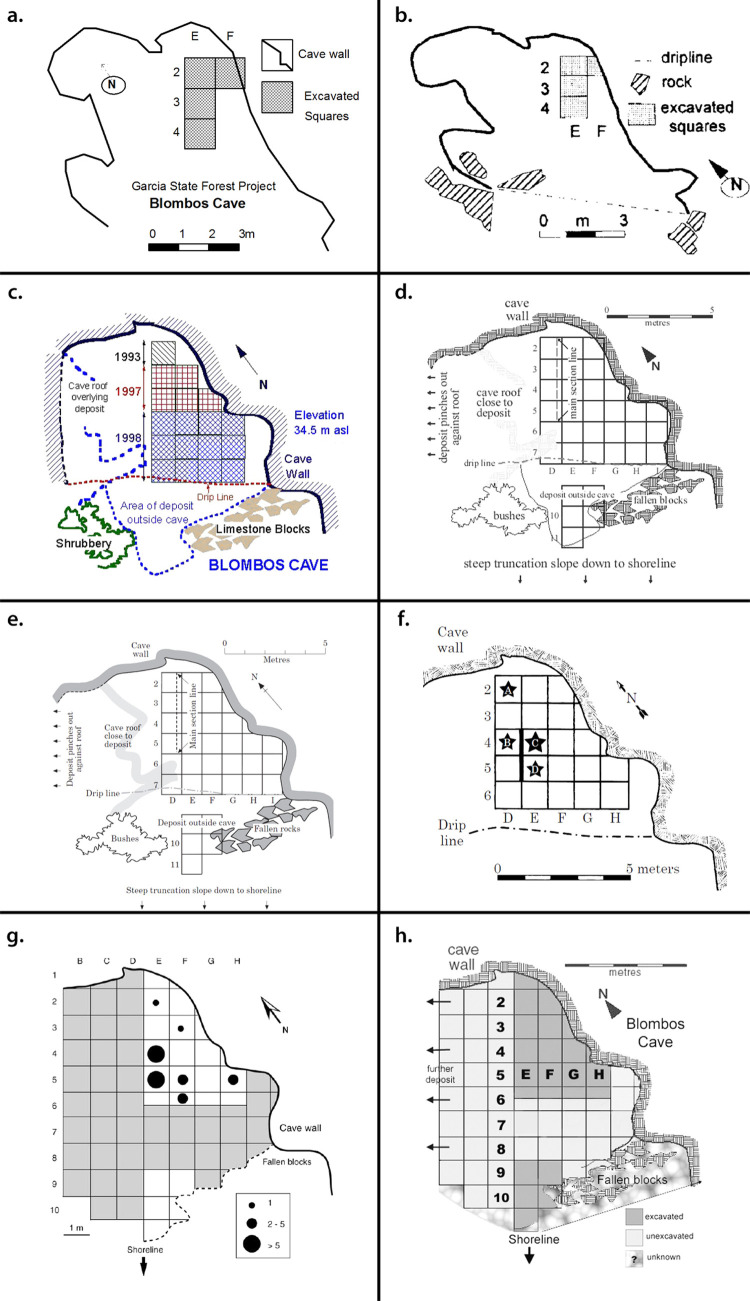
Various iterations of the Blombos Cave site plan. The extent of the depiction and the shape it takes changes over time but has remained limited in detail and information. (A) Blombos Cave excavation notes, 1992; (B) [55]; (C) Blombos Cave excavation report, 1998; (D) [54]; (E) [61]; (F) [56]; (G) [57]; (H) [58] This compiled figure from Haaland 2012 [59].

**Fig 8:**
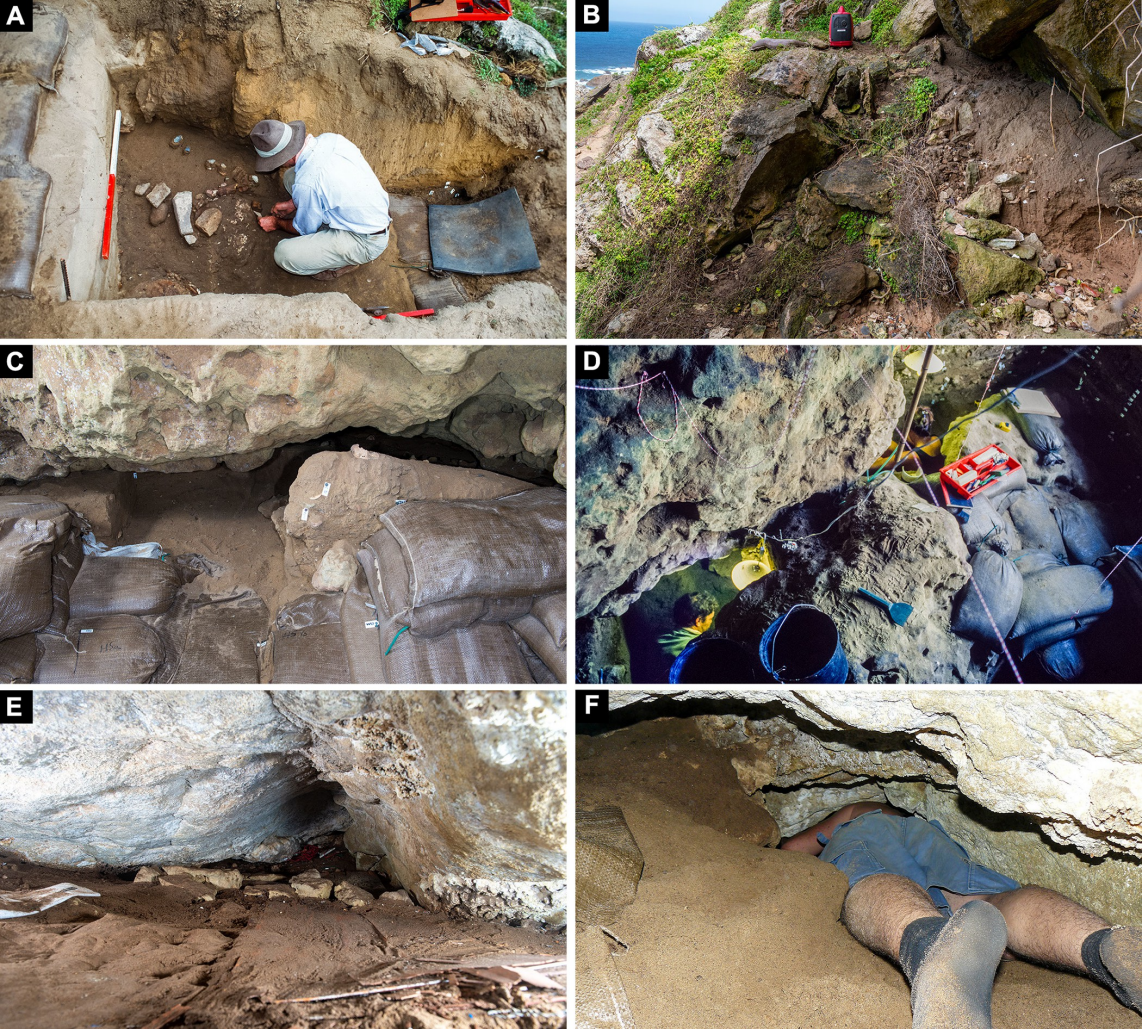
Disconnected areas surrounding the main trench. A number of additional areas have been investigated at Blombos Cave, but their relationship to the main excavation trench is difficult to establish due to spatial separation, visual obstruction and lack of stratigraphic connection. These areas include (A) the talus trench, (B) exterior sediment washout, (C and D) inner chamber and (E and F) western portions of the site which are difficult to access.

The karstic morphology of BBC makes it difficult to map. Although several attempts have been made over the years, none have led to the production of complete or accurate maps of the cave’s topography, morphology, or stratigraphy (Fig 7). Previous mapping efforts have been directed towards the main interior excavation trench, meaning that the exterior talus trench, inner chamber and other peripheral areas have not previously been documented in high resolution or in 3D (Fig 8).

**Fig 9:**
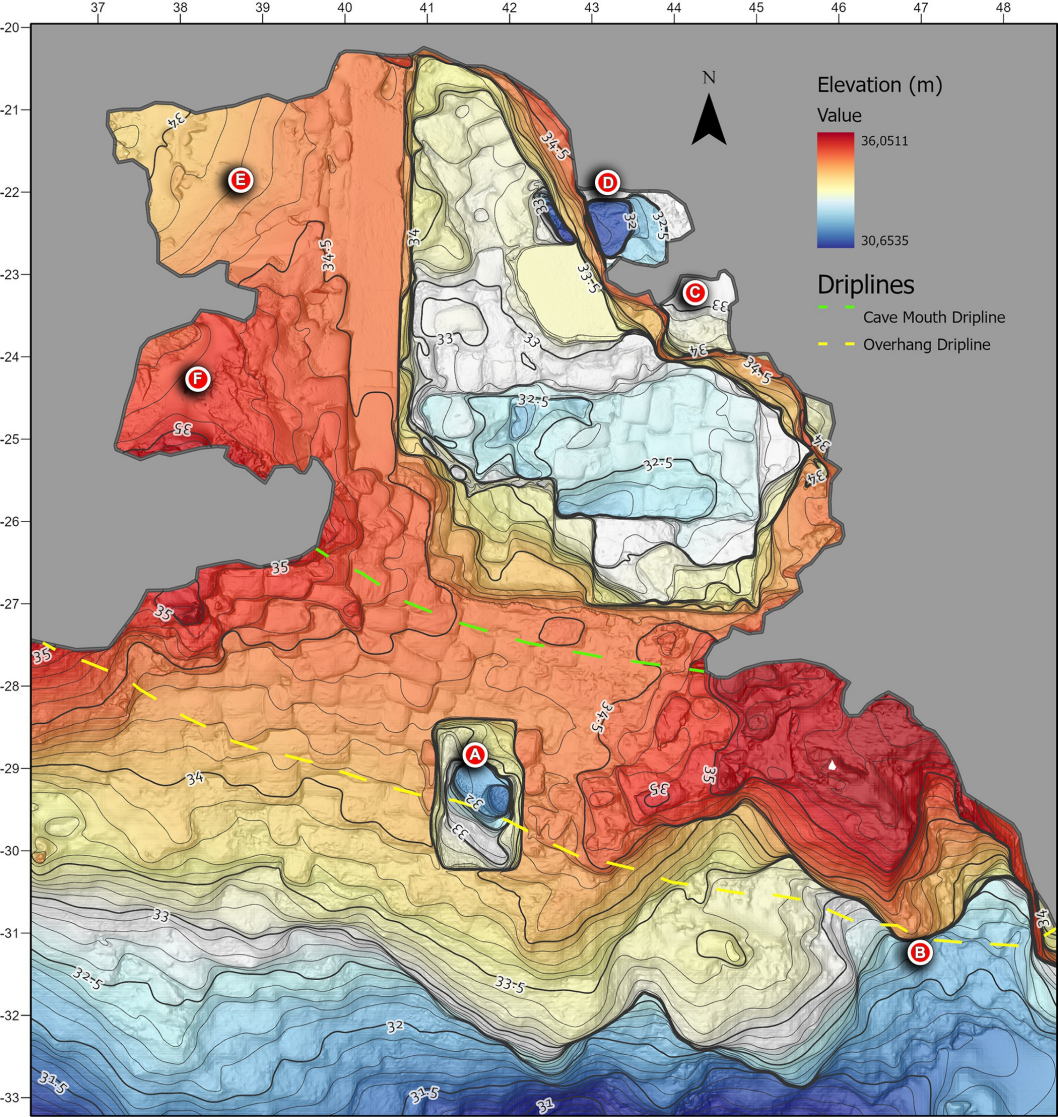
The currently most extensive and detailed topographic plan of Blombos Cave. The plan was produced using a combination of several photogrammetric datasets. Unlike (Fig 7) previous plans, this iteration shows the caves full visible (2020) extent including several previously unconnected areas of the site: (A) The talus trench excavated in 1999, (B) archaeological sediment washout, (C and D) the inner chamber and (E and F) the unexplored areas in western part of the cave (see Fig 8).

To create a more comprehensive and updated site overview of BBC, we used the photogrammetric models of the cave walls, ceiling, and talus in combination with the excavation trenches to generate a complete 3D model of the site’s interior and exterior surfaces (Fig 9). We used the same models to create a high-resolution digital elevation model (DEM) covering the entire exposed interior surface and the talus. The DEM was later imported into a GIS software (ArcMAP 10.7) where it was used to create a topographically correct planar site map. To allow for a complete evaluation of the BBC stratigraphy across the entire site and talus, we use the 3D reconstructed section profiles from different seasons to generate two complete master-sequences. One master-sequence runs on a North-South axis while the other runs on an East-West axis of the site’s local grid system. It was not possible to reconstruct the East-West section using sub-models from one single axis (see section entitled Morphology and stratigraphy). Instead, section profiles from multiple East-West axes were compiled to generate a complete stratigraphic sequence. This was done by generating georeferenced orthomosaic images based on the section profile models. In turn they were imported into the GIS solution, combined, analysed, and vectorised.

#### Visualisation of single-context occupation surfaces excavated over multiple excavation seasons

**Fig 10:**
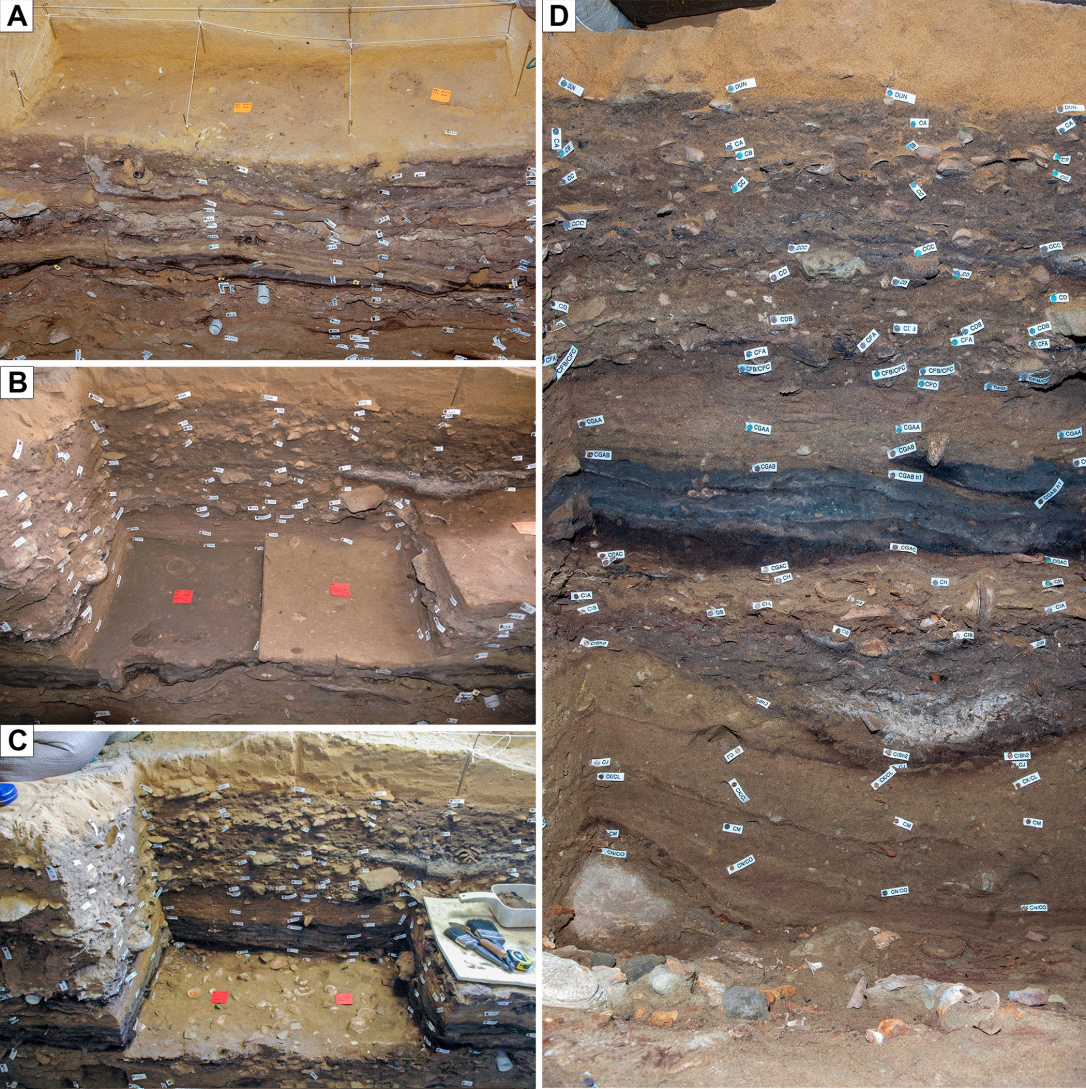
Only limited parts of stratigraphic surfaces and profiles are normally exposed at the same time. (A, B, C) Due to the gradual excavation of BBC, structured by 0.5 x 0.5 m quadrants, (D) large vertical or horizontal exposures of stratigraphy have only occurred rarely during work at the site. Consequently, photogrammetric reconstruction of site stratigraphy has the potential to yield new insights into the record preserved at Blombos Cave.

To document the unusually rich, complex, and intact MSA deposits in detail, the excavations at BBC have been time consuming, meaning that single-context occupation surfaces, originally stretching across the entire cave floor, have been excavated and documented over the course of several field seasons. Consequently, complete spatially/temporally continuous occupation surfaces have never been visible at any stage in the excavation process, making reconstruction and assessment of these surfaces challenging (Fig 10). Retrospective photogrammetry can overcome this challenge by allowing the digital reconstruction of more continuous occupation surfaces that were excavated separately (see section entitled Visual and spatial contextualization of archaeological features, surfaces, and material).

#### Visualisation and analyses of the vertical distribution of MSA artefacts: Combining analogue and digital plots with 3D reconstructed section walls

Recording the 3D location of archaeological artefacts larger than 2 cm (colloquially referred to as *plotted material* or *plots*) has been standard procedure in BBC since 2002. However, it was not until 2011, the first season in which a total station was used, that plots were recorded within a single site-wide coordinate system. Prior to this, plots were recorded using an analogue square specific local XY coordinate system with a site wide Z coordinate provided using a water level. For this reason, it has been challenging to create complete distribution maps showing the location and frequency of plotted material throughout the stratigraphic sequence. In this study, analogue plots were converted to make them compatible with the site’s current digital coordinate system. Conversion from analogue to digital was done by extracting coordinates, from reconstructed 3D models, of recognisable points that had originally been recorded using the analogue (square specific) system. These coordinate pairs were then used to transfer the remaining analogue plot coordinates into the site-wide digital system. These data were then combined with the born-digital (data created directly in a digital format) artefact plots and visualised in combination with the reconstructed surfaces of related quadrant layers and section profiles in ArcGIS Pro. The resulting model allows us to see spatial clustering of artefact plots even where occupational phases slope and are therefore not amenable to being projected onto a section profile.

Data were also visualised in 2D by creating an orthomosaic image of the BBC southern section wall covering the MSA stratigraphic sequence. This orthophoto was used in ArcGIS Pro as a backdrop onto which we placed the projection of 2D artefact plots recorded from the 50x50 cm quadrates immediately behind this section wall. From this basic map configuration, we further visualized the distribution of artefacts throughout the MSA sequence in different ways using multiple analytical (spatial) units and procedures, including: (1) visualisation based on the spatial boundaries of quadrant and archaeostratigraphic layers; (2) visualisation of plotted data normalized by sediment volume information; and (3) visualization based on geostatistical processing of the plotted data using kernel density maps and point cluster distribution analysis.

## Results

### Selection of archive photographs for 3D reconstruction of the site, section walls and excavated surfaces

**Fig 11:**
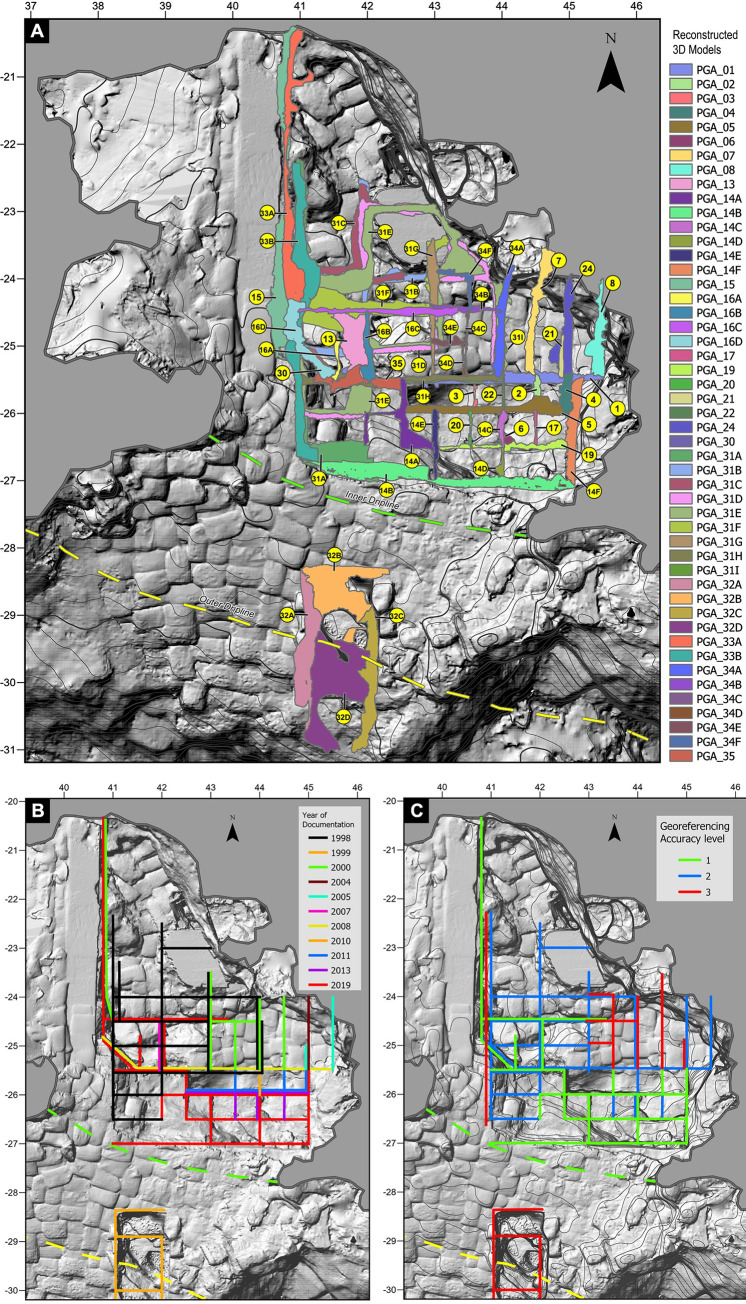
Overview of reconstructed profile sections. (A) 31 individual image sets were used to create 3D models of full or partial profile sections excavated from 1998 to 2019. These reconstructed sections cover about 85% of the excavated area. (B) The older excavations were located towards the rear of the cave while more recent excavations progressed towards the southeast. Panel C shows the distribution of sections by accuracy level. PGA_36 to PGA_42 are not displayed in this figure as they are section surface models. The grey (plan view) backdrop is the general site model PGA_43.

After digitalizing and organising the BBC photographic archive (**Table** 1) and reviewing the optical quality of the photographs (Fig 6), 43 image sets, each documenting an area of the cave but not necessarily consisting of photographs captured at the same time, were identified, and selected for photogrammetric processing. Of the 43 sets, 12 would later be discarded because better image sets covering the same area were identified and compiled. Typically, this occurred with early digital photographs, where digitised slides yielded higher quality models (precise surface, sharper texture resolution, more complete section model, better alignment). From the 31 remaining sets, 59 individual 3D models were generated (Table 2 and Fig 11). 23 of the 59 models were created using analogue photographs (slides) that were captured between 1998–2000 (n = 742). The remaining 35 models were created using digital photographs captured between 2002–2019 (n = 5027).

The 3D models can be classified into three broad categories: section walls (n = 51, Fig 11A), excavation surfaces (n = 7) and general site model (n = 1). In this paper we have primarily focused on reconstructing the interior and exterior trenches and their section walls. A smaller number of excavation surface models were also generated. However, since these are generally exposed for a shorter period, they are less frequently photographed.

**Fig 12:**
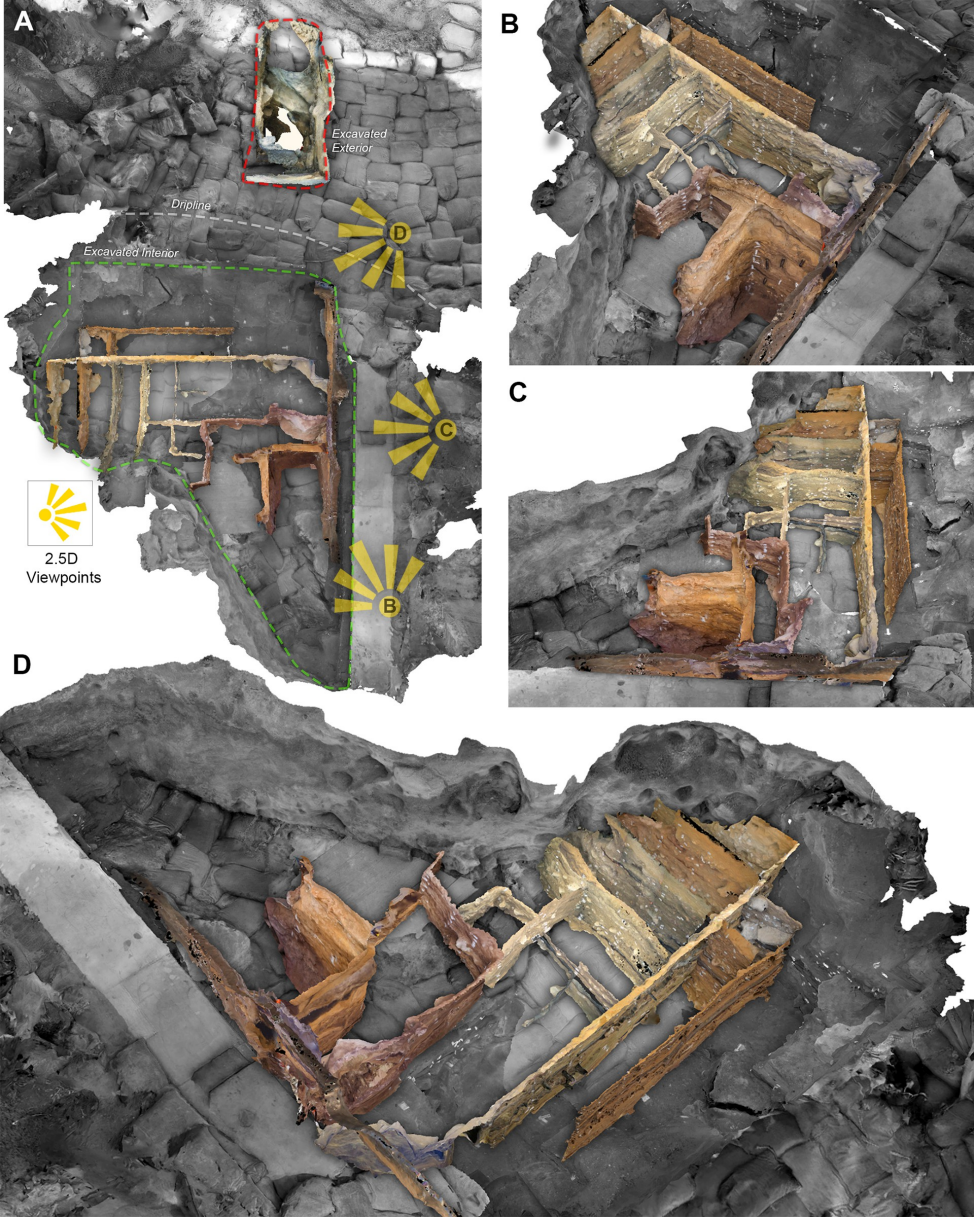
Overview and perspectives of the reconstructed section profiles. The finished 3D models of stratigraphic section profiles can be freely combined and studied in a GIS solution. In this figure, A series of 3D section models (coloured) from across the reconstructed portion of the site are combined with a model of the excavation floor as observed at the end of 2019 (grey). (B, C, D) The nature of this 3D data allows us to rotate and study the combined data from any position and angle. (A) The inset shows a plan view of the site and illustrates the perspective from different viewpoints (B, C, D).

A complete 3D overview of the cave and excavation trenches is provided in Interactive 3D Models 1 (S1 File) and 2 (S2 File). Model 1 shows the general topography of the cave site as well as the full extent of excavation (as of March 2020). In 3D Model 2, which is based on 3D Model 1, we have included most of the reconstructed section walls (some models were excluded to enhance visibility). We estimate that around 85% of the total area excavated in BBC are either fully or partially covered by one or more of the retrospectively produced photogrammetric models (Fig 11). A print-friendly 2.5D view of 3D Model 1 and 2 is provided in Fig 12.

### Accuracy evaluation

**Table 3** and Fig 11C provides an overview of all the image sets used to create the 3D models presented in this paper, along with the source of their GCPs (accuracy level 1–3, see section entitled: Georeferencing the 3D models and evaluating their accuracy). This table also provides the root-mean-square (RMS) deviation for each individual model, as well as the cumulative RMS for the models using secondary and tertiary GCPs.

**Table 3:** Model accuracy.

Dataset	Number of reference points	Estimated Model error (mm)	Estimated Accumulated error (mm)	Accuracy Level	Year
**PGA_001**	10	15.18	18.50	2	2007 and 2008
**PGA_002**	38	6.34	6.34	1	2011
**PGA_003**	10	3.75	3.75	1	2011
**PGA_004**	8	3.91	3.91	1	2011
**PGA_005**	57	4.50	4.50	1	2011
**PGA_007**	36	17.34	25.36	3	2000
**PGA_008**	10	9.80	14.41	2	2005
**PGA_013**	6	3.71	11.20	2	2007
**PGA_014**	30	2.74	2.74	1	2019
**PGA_015**	10	2.96	2.96	1	2019
**PGA_016**	4	0.68	0.68	1	2019
**PGA_017**	10	1.48	10.67	2	2013
**PGA_019**	16	1.03	1.03	1	2019
**PGA_020**	10	1.41	10.66	2	2013
**PGA_021**	10	6.15	19.49	3	2005
**PGA_022**	13	6.76	8.12	2	2010
**PGA_024**	5	2.58	10.88	2	2002
**PGA_030**	4	0.68	0.68	1	2019
PGA_31A*	12	16.07	19.23	2	1998–1999
PGA_31C*	12	15.95	19.13	2	1998–1999
PGA_31D*	12	15.75	18.96	2	1998–1999
PGA_31E*	12	14.85	18.22	2	1998–1999
PGA_31F*	12	15.78	18.99	2	1998–1999
**PGA_32**	6	15.16	24.49	3	1999
PGA_33A*	8	13.94	13.94	3	1998–2000
PGA_33B*	7	10.54	10.55	3	1998–2000
**PGA_34**	6	0.65	18.51	3	2000
**PGA_35**	31	2.61	2.61	1	2019
**PGA_36**	11	7.25	7.25	1	2011
**PGA_37**	9	5.20	5.20	1	2010
**PGA_38**	13	3.44	3.44	1	2011
**PGA_39**	9	7.30	7.30	1	2010
**PGA_40**	7	1.42	1.42	1	2011
**PGA_41**	10	3.51	3.51	1	2011
**PGA_42**	10	3.09	3.09	1	2011
**PGA_43**	14	10.57	10.57	1	2013

This table provides an overview of the spatial accuracy estimated for each individual 3D model based on the deviation from the Ground Control Point (GCP) coordinates used to geographically reference the respective models. For models that are not directly referenced an accumulated error is provided. The models accuracy is defined into three levels.

18 of the 3D models were directly referenced (Level 1 models), 12 where indirectly referenced (Level 2 models) relying on GCPs from one of the 18 Level 1 models. The remaining 6 3D models (Level 3 models) where in turn referenced using GCPs collected from one of the 12 Level 2 models. The range of spatial uncertainty for the models is 0.68 mm to 25.36 mm.

**Fig 13:**
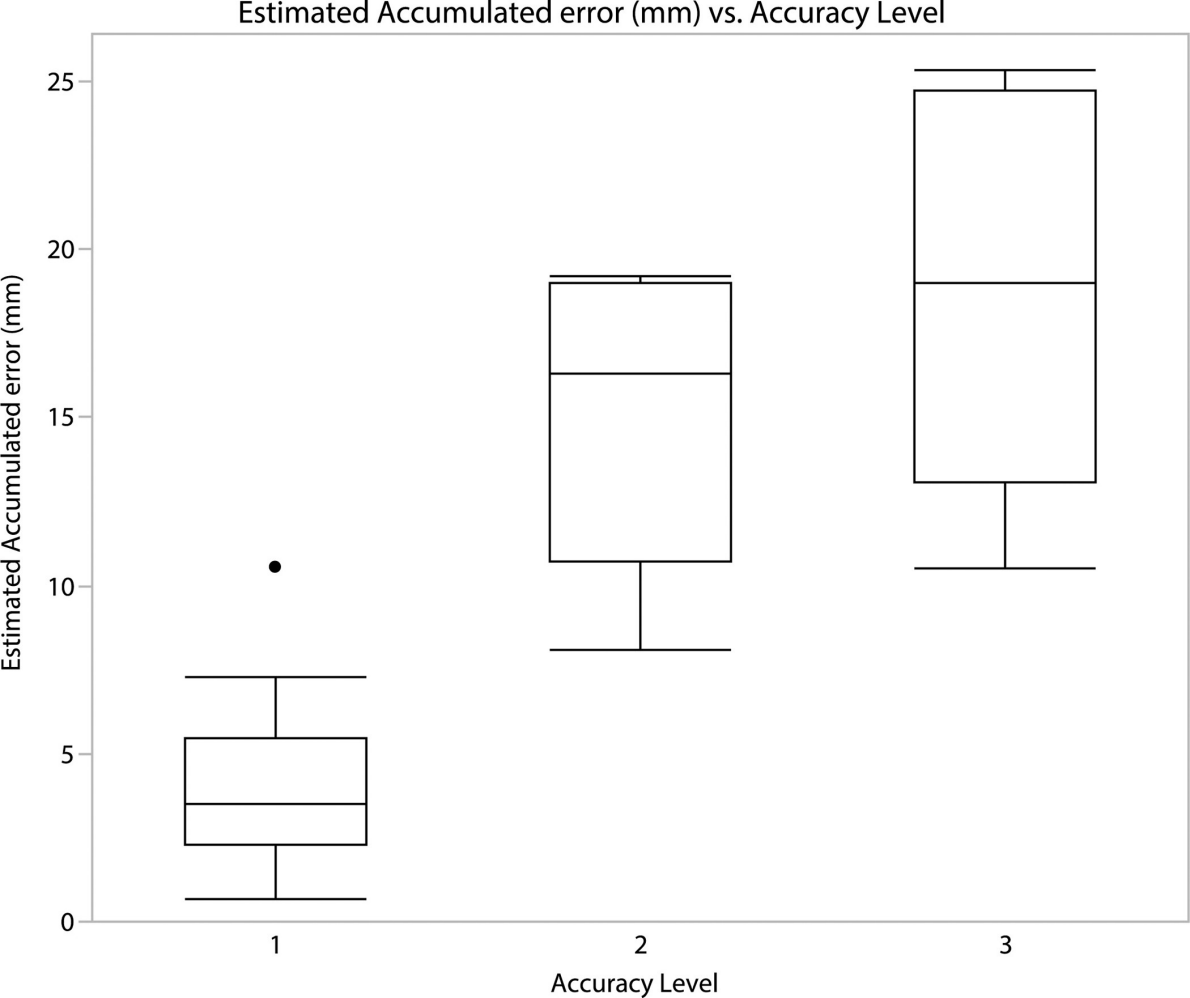
Box and whisker plots showing the range of estimated accuracy for the directly and indirectly referenced models. The majority of directly referenced models have an estimated spatial uncertainty of less than 8 mm. The outlier represents the large site model recorded in 2013 (PGA_43), which covers the entire site. The large scale of this model, and even distribution of GCPs, lead to a higher spatial uncertainty. Because different level 1 models have been used to reference level 2 and 3 models, the uncertainty ranges of the latter overlap.

Level 1 3D models were constructed using image sets intentionally captured either for orthophoto or for photogrammetric reconstruction (2011–2020). Level 2 models were primarily generated from photographs captured by various digital cameras but predating the introduction of a total station (2002–2010). Most level 3 models were produced using digitalised slides i.e., using images captured with an analogue film camera (1998–2000). Analysis of the whole dataset by GCP level shows the effect of cumulative uncertainty. Level 1 models have lower estimated uncertainties than Level 2 models which are in turn more accurate than Level 3 models (Fig 13). However much of the uncertainty associated with level 2 models stem from an anomalously large uncertainty of a single level 1 model, PGA_43. This model is the general site model recorded in 2013 [38], it is both large and complex and was georeferenced using relatively large targets (1cm circular stickers as markers) acting as GCPs. These targets where in part recorded at a high angle, reducing the total station and its operator’s ability to record the target’s centre, additionally the surface where some of these targets where placed, was photographed at a greater distance resulting in a lower surface resolution (pixels per centimetre). The other level 1 models presented in this paper record single, relatively small sections or surfaces, and were georeferenced using small targets (nail heads or specially made targets (< 5 mm)) and were photographed at a closer range (resulting in a higher surface resolution). This allowed for a more accurate recording and placement of GCPs, which resulted in a lower estimated uncertainty. Consequently, the apparent step change between the accuracy of level 1 and 2 models is an artefact of the fact that most level 2 models are derived from the single relatively imprecise level 1 model, PGA_43. Consequently, at most sites the precision penalty incurred using level 2 and 3 models is lower than that implied by visual inspection of Fig 13. Analysing the results achieved using different cameras indicates that a general increase in accuracy is observed with improving camera specifications. However, the purpose for which images were captured as well as the scale of the subject recorded is also important. For example, although the mean uncertainty calculated for level 1 models using images captured with the Nikon D4 is lower than that achieved using the D700, the range of results for the latter camera is lower. This is because most of the D700 images used were captured to produce a high-resolution orthorectified photomosaic of a relatively small area. This unintentionally yielded a dataset amenable to photogrammetry, owing to a high level of overlap and detail captured. Conversely, the level 1 models produced using D4 images have a larger range of uncertainties owing to the large uncertainty of model PGA43 previously discussed. Overall, the most accurate results are achieved within level 1 models using better specification cameras. However, the penalty incurred using archival photographs and level 2 and 3 referencing does not preclude the production of serviceable models.

### Cross seasonal site wide analysis

#### Site topography

Fig 9 shows the spatial relationship between the primary excavation and other areas of investigation such as the exterior trench, cave talus and inner chamber (Fig 9A, 9B and 9D). Also shown are the areas of the cave preserved for future investigation (Fig 9C, 9E and 9F). Besides providing a more comprehensive overview of the site than the traditional plans shown in Fig 7, it also differs by showing the actual extent, shape and depth of the excavation area as opposed to the intended extent adhering to the sites’ grid coordinate system. For example, section walls are intended to be straight, and would be represented as such in a traditional plan, but the 3D model faithfully depicts and “wobbles” that occur.

#### Morphology and stratigraphy

**Fig 14:**
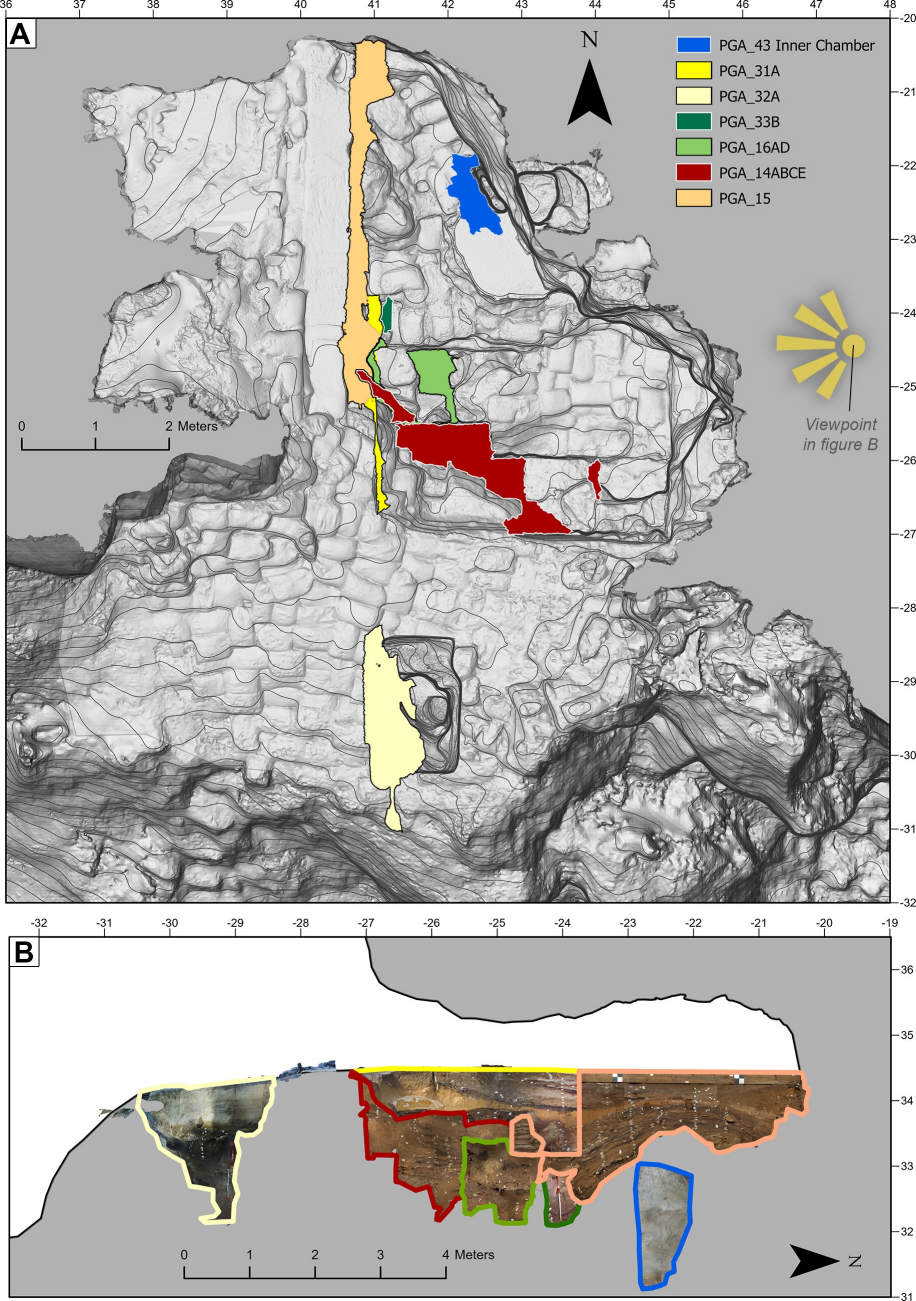
A north-south stratigraphic cross section of Blombos Cave. (A) Multiple spatially referenced orthophotos were generated using a selection of 3D models from different years. (B) By combining these we created a high resolution, sitewide, multi seasons stratigraphic overview along the Y axis of BBC. While this overview does not provide a fully connected stratigraphic overview, it nevertheless provides the most comprehensive stratigraphic overview to date.

**Fig 15:**
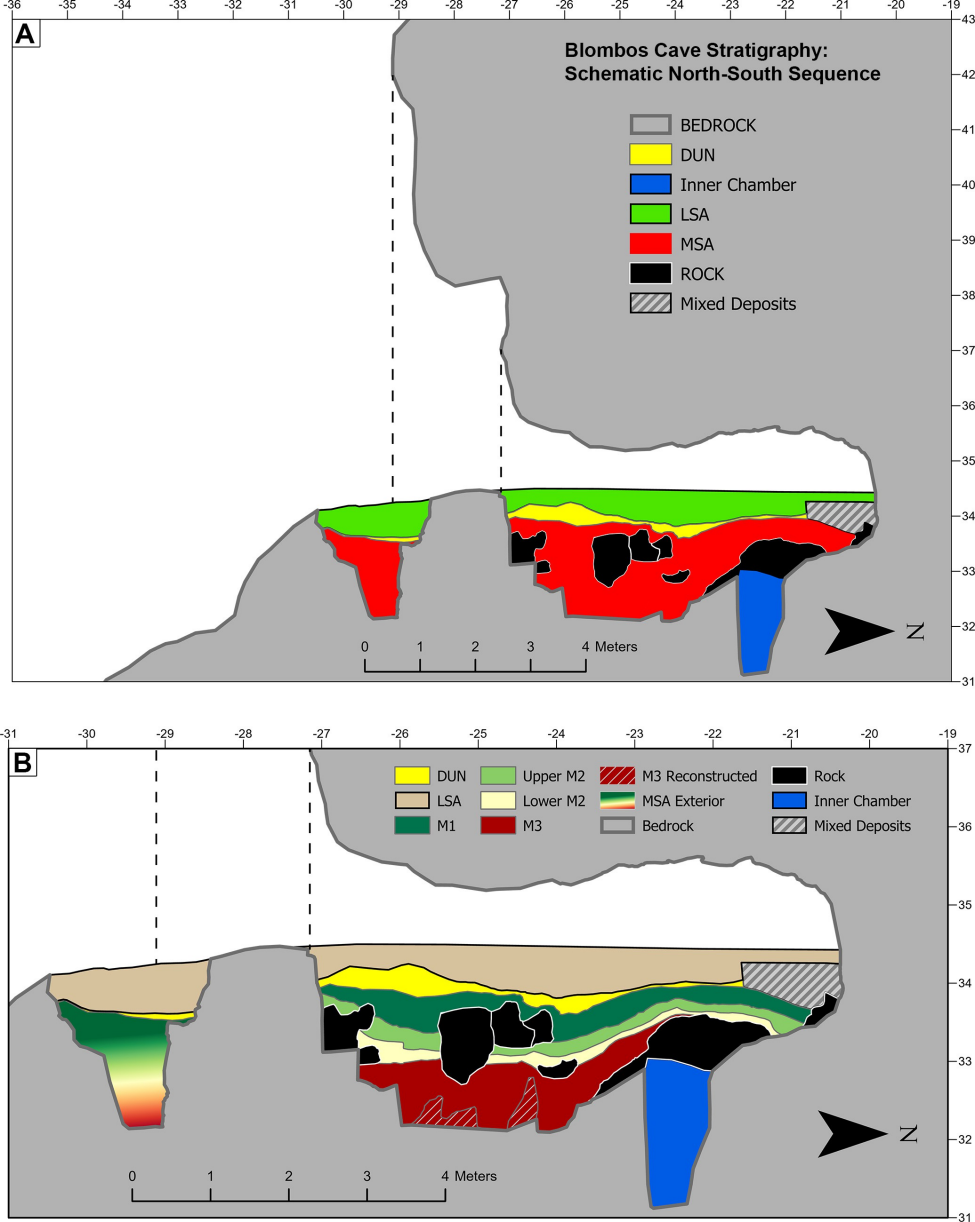
(A and B) Schematic overview of BBCs North-South stratigraphy from the back of the cave to the exterior talus trench. This figure was produced using the archive section reconstructions shown in Fig 13 in combination with a larger landscape model that provided the surface of the surrounding landscape. This figure allows spatial relationships between disjointed parts of the cave and surrounding landscape to be established. Because the stratigraphy connecting the main excavation area and the talus trench has yet to be excavated, stratigraphic relationships with the latter were inferred based on depth.

**Fig 16:**
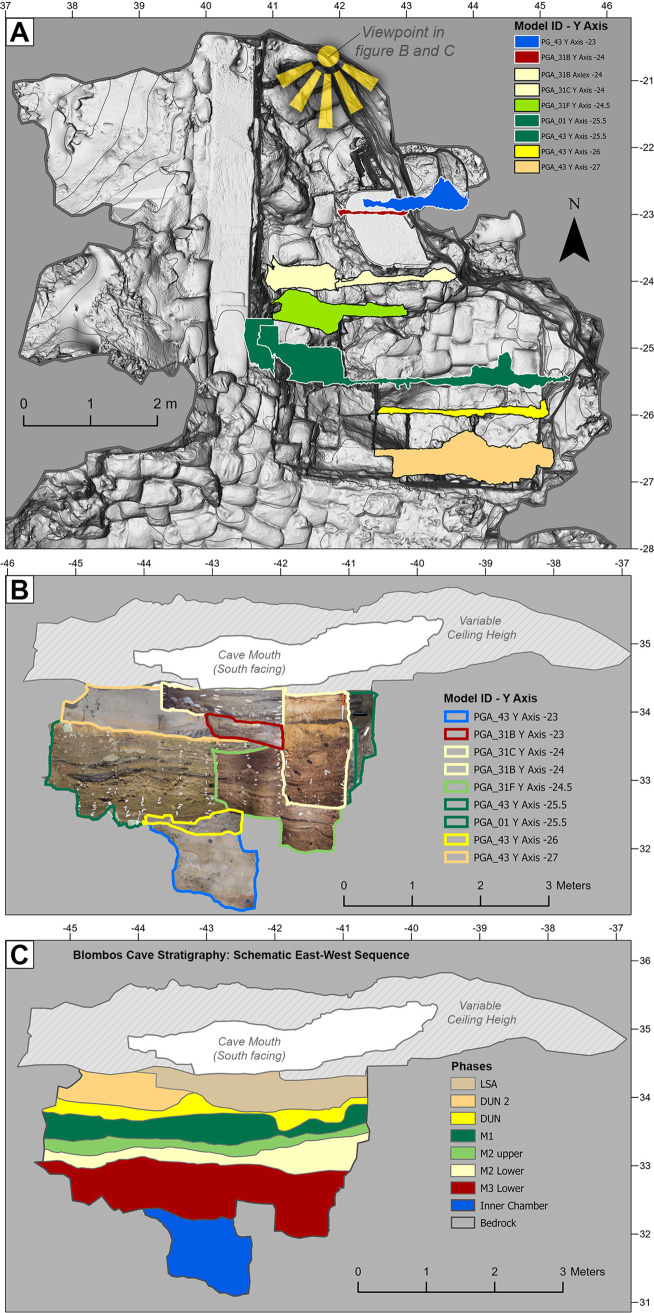
(B and C) East-west overviews of the cave stratigraphy (A) produced by combining a selection of profile sections along the site’s x-axis. Due to the order of excavation, available photographic material, natural shape of the cave and large boulder’s this stratigraphic overview had to combine sections from different points on the sites Y axis. Consequently, panel C thus provides a stratigraphic overview of the sites stratigraphy that has not been possible before.

To allow for evaluation of sediment morphology and stratigraphy, two master sequences were created along the site’s Y (Figs 14 and 15) and X (Fig 16) axes. Fig 14 shows the maximum extent of the uncovered sediments along the site’s Y axis viewed from the east. It was created by combining orthographic images generated from 7 of the photogrammetric datasets. In Fig 14B the main excavation sequence is composed of 3 models (PGA_15, PGA_31A, and PGA_33B) along a single grid line. In combination these models represent a true section which, had the excavation sequence permitted it, could have been observed as presented. Conversely due to the morphology of the site and location of a large rockfall, the lower southern end of the main excavation sequence was constructed from sections located on more easterly grid lines. This yields a composite section, which has never existed, but which represents the full thickness of the archaeological sequence uncovered at BBC. Fig 14B also shows the inner chamber and exterior talus trench which allows depth relationships to be understood between areas that are spatially separate from one another. Based on this composite sequence, a schematic representation of the location of the MSA and LSA deposits was constructed (Fig 15A) with more detailed subdivision into occupational phases presented in Fig 15B.

To create a composite section overview along the X axis it was necessary to combine orthographic images from multiple section models located along the Y Axis (Fig 16A). This was a consequence of the cave’s morphology, the same large rockfall described above and the excavation sequence. As with the lower southern end of the north-south section this yields a composite section that has never existed (Fig 16B). Given the range of different sections needed to create the composite, it yields a less perfect representation of the sequence. This is particularly problematic in the upper portion of the sequence where the LSA (PGA_31C) is underlain by archaeologically sterile sand (PGA_43). At BBC the LSA is primarily located in a shallow bowl like feature towards the centre of the cave, whereas the sterile sand is only present in an East-West photogrammetric section towards the front of the cave in the southeast. A schematic overview of the occupational phases was created based on the composite sequence (Fig 16C). Unlike the North-South overview (Fig 15) this composite sequence was primarily made up of section models that did not intersect. Therefor some interpretive decisions were made. The transition between the LSA (PGA31C) and underlying DUN (PGA_43) units has been smoothed in Fig 16C relative to the transitions in Fig 16B. This is because the elevation of this transition changes in a north-south direction, and therefore experiences abrupt elevation changes from one section model to the next. The result is a subjective interpretation of the transition, but one which more faithfully reflects the nature of this feature as it is observed in individual section models.

#### Reconstructed single context surface

**Fig 17:**
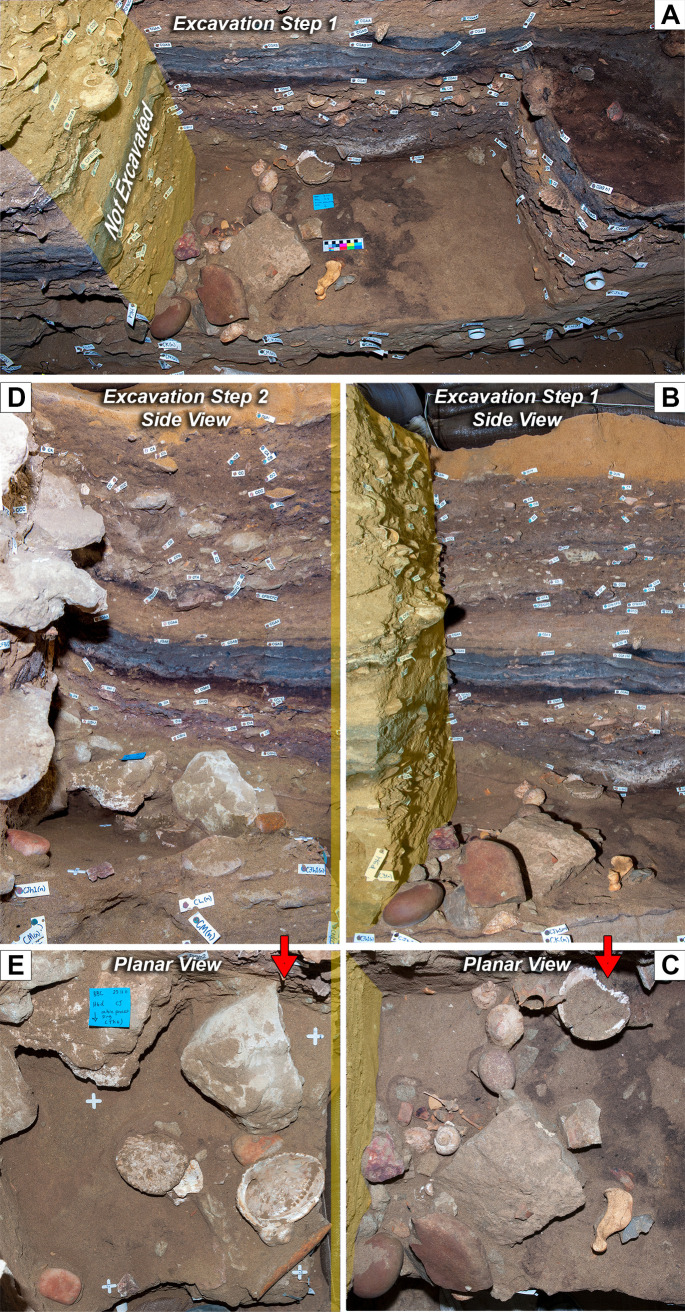
A cluster of artefacts uncovered at different stages of the 2011 excavation season. (A, B, C) The first part of this artefact cluster was documented and fully removed before (D, E) the second part was uncovered. In an attempt to reconstruct the artefact cluster, (D and B, E and C) pairs of still photographs where imposed side by side.

**Fig 18:**
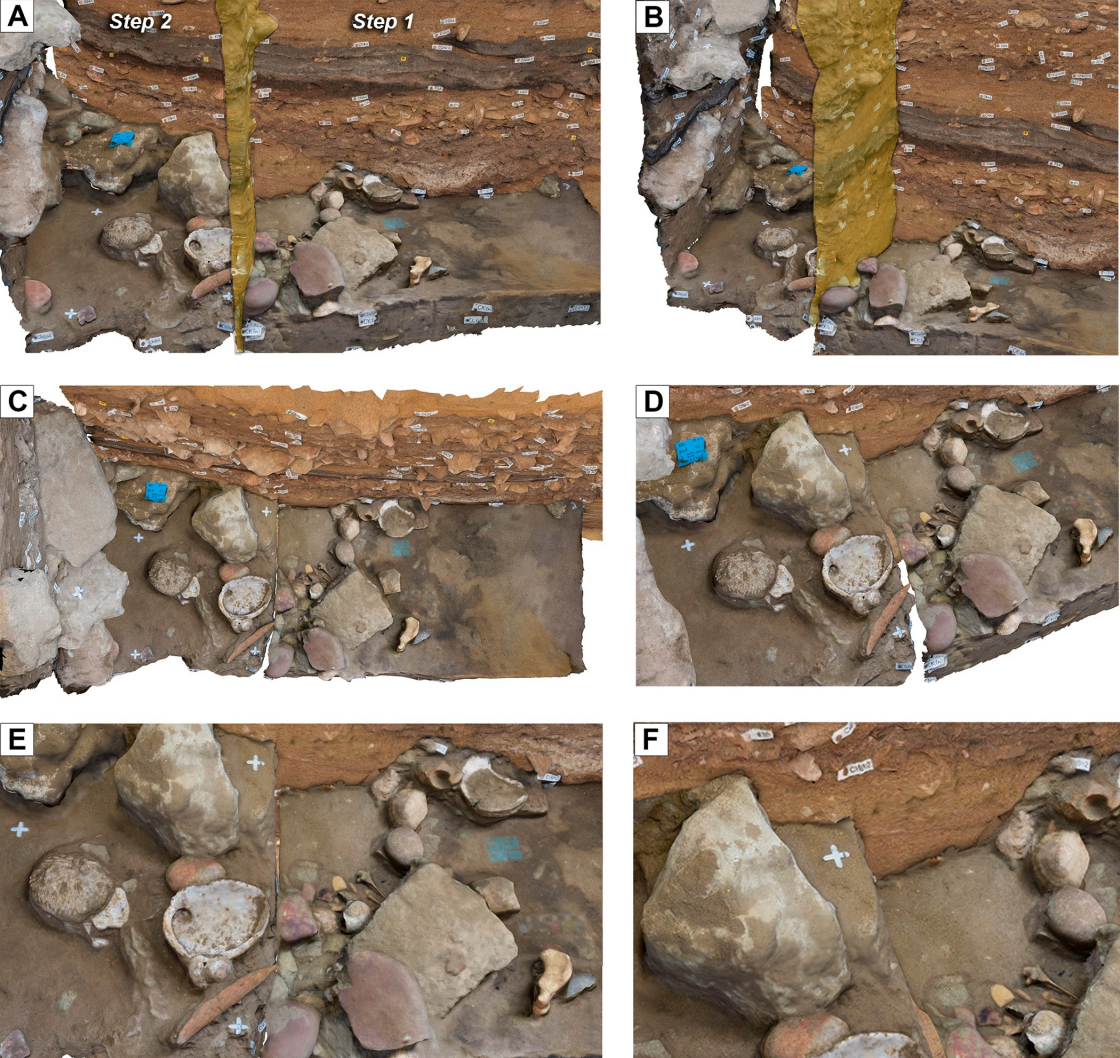
Reconstructed artefact scatter surface. Using models PGA_2, 4, 5, 40 and 41, the artefact scatter found across two separately excavated quadrants of the layer CJ was reconstructed. Panels A and B show surface CJ, with the vertical orange section profile showing division between quadrants. Panels C-F show the artefact scatter on CJ from multiple angles, illustrating the potential of this model for investigating spatial relationships between excavated objects. Unlike the photomosaic in Fig 16, this 3D reconstruction shows the actual measurable spatial relation between the material with a known error margin. This is because the eastern quadrant’s artefact scatter was photographed before layer CJ had been completely excavated.

A portion of layer CJ was reconstructed to illustrate the value and risk of photogrammetry in understanding individual contexts. This area was excavated as three 50X50 cm quadrats, only two of which were exposed simultaneously (Fig 17A). This is particularly problematic for layer CJ which contained a cluster of artefacts divided between two quadrants excavated two weeks apart in 2011 (Figs 17B–17E, 18A and 18B). The application of retrospective photogrammetry allowed for the reconstruction of this area of the surface, yielding an accurate 3D reconstruction of the artefact cluster (Fig 18C–18E). Comparison of this 3D reconstruction with the photographs (Fig 17C and 17E) initially implies the same relationship between quadrant surfaces. However, abutting edges of quadrants do not meet perfectly as is evident from the gaps between models in the reconstruction (Fig 18E and 18F). These gaps, which are as large as 7.5 cm in places, exceed the uncertainty calculated for the individual models (PGA_40, 1.42 mm and PGA_41, 3.51 mm, Table 3). Consequently, the gaps were initially interpreted to result from the difficulty of defining surfaces during excavation. For example, Fig 18F shows that the right-hand quadrant looks like it was overdug relative to that on the left. The surface on the left-hand quadrant appears to have been defined by the linear feature observed in the back wall of the right hand quadrant. Subsequent reference to field notes, however, revealed that in fact the image set used to create the model was taken prior to completion of digging the quadrat. Consequently, while the 3D reconstructions give a better representation of the spatial relation and orientation of the artefacts found across the quadrant layers then that of the point plots and still images, it does not represent the actual base of the stratigraphic layer. This demonstrates that detailed site knowledge and written records are there for important to ensure a correct understanding of what a generated model represents.

#### Visualised vertical distribution of artefacts

**Fig 19:**
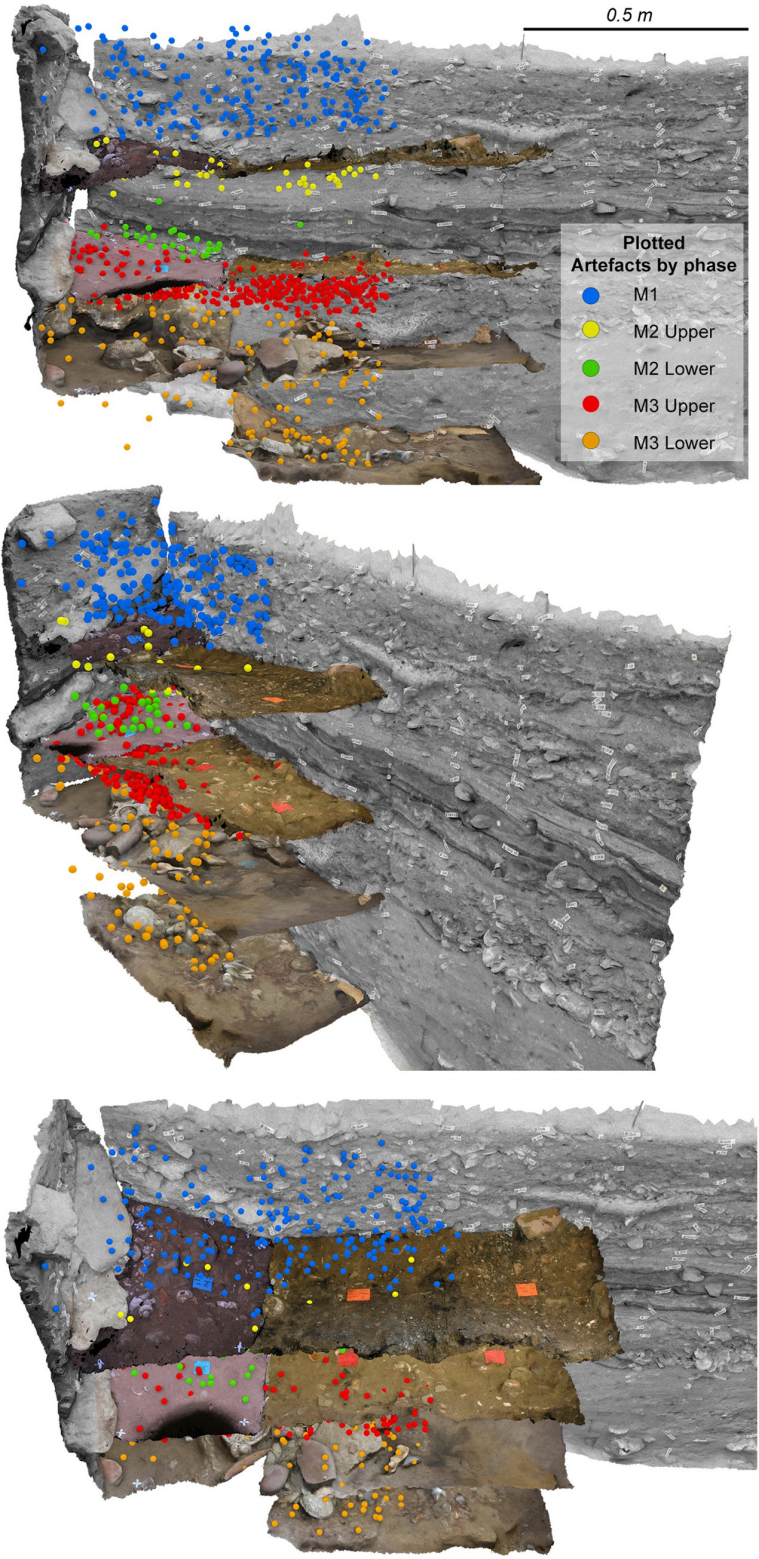
3D model of individual plotted artefacts shown with selected section profiles and quadrant layer surfaces. The point plots for M1 to M3 upper in the right half were recorded using analogue techniques whereas those on the left were recorded digitally using a total station. The analogue points were plotted within a quadrant specific coordinate system, wheras the digital coordinate system is site wide. The three panels show the same data from different angles and illustrates the compatibility achieved when combining the different datasets.

Fig 19 shows the 3D visualisation of the analogue and digital plots in combination with related layer surfaces and rearward bounding section profiles. At a coarse scale, the vertical separation of artefact plots by phase gives confidence that our combination of analogue and digital data yields realistic results. Analysis indicates that clusters result from real concentrations of artefacts rather than the concatenation of unrelated artefacts with a common Z coordinate as could occur in a 2D representation of sloping sediments.

**Fig 20:**
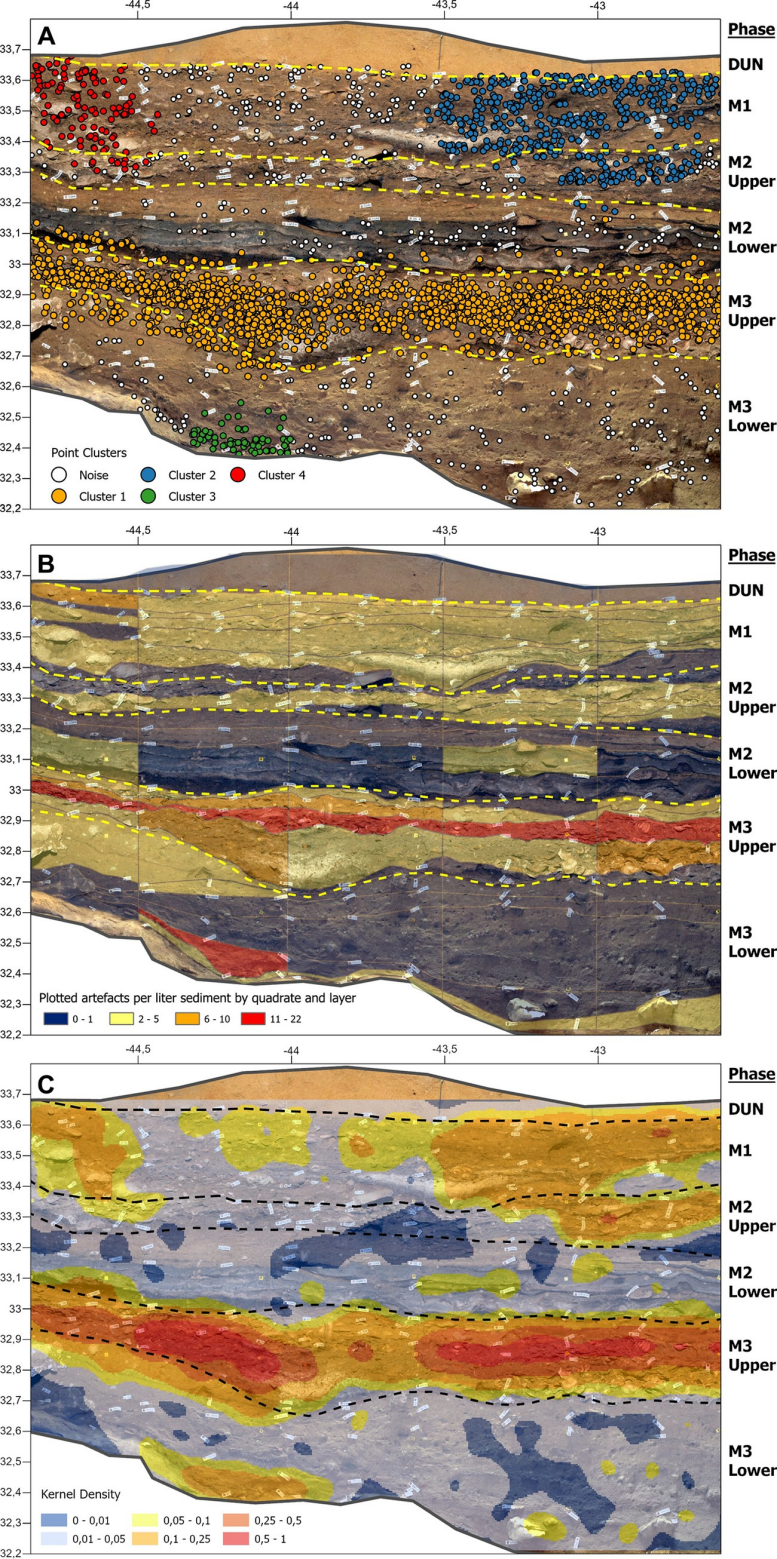
Different ways of visualising artefact distribution by combining plotted artefacts and reconstructed section profiles. Plotted artefacts recorded with digital and analogue methods combined with an orthographic image of the quadrants southern section profile analysed and visualised using ArcGIS Pro 10.7. (A) A cluster analysis was used to identify 4 artefact clusters. (B) The number of artefacts recorded per litre of sieved sediments for each quadrant layer. (C) A kernel density map of the plotted artefact data from panel A.

Fig 20A shows clustering of plotted artefacts projected onto the stratigraphic section profile and defined occupational phases. Point cluster analysis conducted in ArcMAP 10.7 suggests the presence of 4 clusters. Fig 20B shows artefact concentration per litre of archaeological sediment visualised by unit and quadrant. Fig 20C is a kernel density map of the plotted artefacts projected onto the orthographic section profile overlay by the occupational phases. The data underlying this visualisation are those presented in Fig 20A.

## Discussion

In the following sections, we will first discuss the challenges and limitations encountered when applying a retrospective photogrammetric workflow to the BBC archive photographs. We will also evaluate the extent to which we managed to reconstruct the site at different stages of excavation. We will also discuss how the 3D models may serve as an analytical and contextual backdrop that enables us to spatially inter-link archaeological material with its surrounding field context. Finally, we will consider–based on the case studies from BBC, the potential, and limitations of retrospective photogrammetry in general and provide suggestions for best practice guidelines.

### Challenges, limitations, and extent of site reconstruction

#### Operational challenges and limitations related to data availability

At the start of this study, the BBC photographic archive had not been consistently organised or labelled, and the photographs were also stored in fundamentally different formats (analogue slides and digital files). Locating, digitalizing, compiling, organizing, and selecting photographs relevant for 3D site reconstruction, required both time and in-depth knowledge of the excavation history of the site. Specifically, the need to use photographs captured during different seasons, made it essential to develop an intimate understanding of the site’s particular configuration at various stages of excavation. This understanding, which was enhanced by gradual familiarisation with the archive, improved our ability to recognise relevant images. From the early stages of excavation where relatively few photographs are available, exclusion criteria such as lighting, motion blur, focus etc. cannot be applied as stringently as for later stages, from which abundant images were available.

In our retrospective photogrammetry workflow, selecting images that positively contributed to the reconstruction of a section wall or an exposed cave surface was not always straightforward. This was particularly the case for those photographs that were not taken with photogrammetry in mind. In these photographs, people and objects not related to the excavation had to be manually masked before they could be used for 3D model creation. The analogue photographs also posed some specific challenges. These related to their relatively low numbers, their limited variety of camera angle, the lack of overlap between each photograph, as well as the fact that information on the lens and camera parameters were not recorded. The latter two factors most frequently prevented automated 3D model generation. In the case of low image overlap, common features tend to occur in the periphery of images, where lens distortion is most pronounced. Consequently, manual identification of shared features was necessary.

In the case of dataset PGA_07 and PGA_32 a trial-and-error process was required to identify confounding images. For example, without the removal of confounding images, dataset PGA_07 yields a markedly concave model, which results in a low accuracy of the GCPs used to align it and is clearly at variance with both the grid system of the site and visual inspection of individual images. Repeated iterations of model generation, excluding potentially confounding images, yielded a progressively straighter section profile. This highly time-consuming process was repeated until an acceptably low deviation from confidently located GCPs was achieved. The most likely cause of confounding images in this case is a variety in focal length which directly affect the distortion of an image and is not available for the analogue images. In such a case the photogrammetric software assumes a consistent focal length across the dataset and may achieve an imprecise result due the inconsistent focal length causing variable distortion between the images [22].

The problems identified above are likely to be most pronounced in cramped sites like BBC where the number of available camera angles is limited.

#### Limitations related to spatial accuracy and textural resolution

We have identified two main factors that determine how useful the 3D models made from archive photographs can be: textural resolution and spatial accuracy. The former determines what we are able to recognize visually while the latter limits the confidence we can place in the data’s ability to correctly show us the actual position of the modelled material. For example, textural resolution is an important factor in allowing the identification of individual archaeological features, materials or layers and determining their distribution or morphology across the site (Fig 6). When the textural or optical resolution is too low, it prevents any meaningful visual inspection of the model. Our calculations show that the BBC models have a spatial accuracy range between 0.68–25.4 mm (Table 2), with a mean of 10.1±0.2 mm. The stated uncertainty of points measured using a total station (Trimble Spatial Station VX) is ±2.0 mm, though a realistic achievable uncertainty during excavation will be considerably larger than this due to factors such as the daily positioning of the instrument, switching between direct measurement and the use of a small prism staff when line of sight is obstructed and the use of point locations to represent 3D objects at the site. For practical purposes the mean uncertainty of the 3D models is likely to be consistent with that of the total station data.

#### Evaluating the extent of site reconstruction using retrospective photogrammetry

In earlier site recordings of BBC (Fig 7) the site maps have focused on the excavated areas and their location relative to the site walls. These maps do not show the true shape of the cave or the exact location of the excavation trenches in relation to the drip line, talus, or exterior test trench. Instead, the 2D site maps have depicted a schematic cave outline and an idealized trench layout in the form of a perfect grid. While earlier recordings of site stratigraphy have achieved high visual fidelity and spatial correctness [61], these recordings also fail to provide a complete site overview, showing the true shape and spatial relations between the different areas of investigation. By using archive photographs the site’s morphology, configuration and topography can finally be visualised, spatially documented, and appreciated in ways that were not previously possible. For example, the confined nature of BBC makes the true shape of the site difficult to comprehend during excavation. Similarly, some areas of the site such as the exterior trench, main excavation, and deep sounding (Fig 8), cannot be viewed simultaneously in the field. Therefore, determining the precise spatial relationship between these areas has been difficult until our application of retrospective photogrammetry (Figs 9 and 15).

#### Evaluating the extent of trench reconstruction using retrospective photogrammetry

Prior to this study, less than 30% of BBC trenches had been reconstructed in 3D. Now we were able to successfully reconstruct excavated section walls across ~80% of the excavated area. The completeness of trench reconstruction varies, as not all section profiles are documented in their entirety (from base to top). This is due to both variations in the number of photographs taken, visual obstructions encountered within the sediments (i.e., rockfall) and the practical setup and execution of the excavation (not all angles of profiles are physically possible to cover due to the fragile sediments limiting where a photographer can stand).

Variations in the number of available photographs result from changes in recording strategy, methodology and excavation focus. For example, the lack of reconstruction in the northern part of the cave is caused by the limited number of photographs taken during the early stages of excavation (Fig 11). This was a time when film rolls were expensive, and photographs had to be carefully planned. A final factor in the completeness of the available photographic record comes from the subjective actions of the excavation team deciding how to excavate and which section profiles to retain for thorough photographic documentation. In some cases, a single quadrant (50x50 cm) was completely excavated before progressing to the neighbour, while in other cases several adjacent quadrants were excavated simultaneously (Fig 10A). This meant that in some areas a full section profile was preserved and documented whereas in others it was gradually removed before any substantial vertical section could form.

To summarise, the specific order of the excavation can affect the completeness of the reconstruction in two ways. Firstly, cave morphology and obstructions such as large roof spall or boulders impose physical constraints upon the camera angles available during excavation. Large obstructions also interfered with the planned location of section faces within the excavation grid. Secondly the decisions of the excavators determined which section faces where made available for photographic documentation. For example, where a quadrant was deliberately overdug relative to adjacent quadrants, the front wall does not tend to be well enough exposed for photographic documentation. While the confined and dimly lit space in a cave represents natural limitations to photographic documentation, the subjective decisions made by the excavators during the field documentation process represents a universal factor that will be present in all types of archaeological settings. Universally, a good understanding of the site and the history of its excavation is required to make the most complete and accurate reconstruction.

### Retrospective photogrammetric reconstruction: A starting point for cross-seasonal and site wide analyses

#### Visual and spatial reconstruction of archaeological trenches, section walls and stratigraphy

Combining all 3D reconstructed section walls within a single model (Figs 11 and 12) provides a fully interconnected stratigraphic overview that was never visible at a single point in time. Digitally, however, it is possible to view, explore and evaluate the textured section wall models regardless of when excavation occurred. By tracing the archaeologically defined layers on the 3D reconstructed section walls, we are also able to create vectorized profile views that run across the entire site. This ability is particularly important for a site like Blombos Cave, where multiple recording practises (Fig 5E) have resulted in non-interoperable field records and non-georeferenced site and section wall drawings.

The value of documenting the cave’s stratigraphic sequence is demonstrated in Figs 15 and 16. For the first time during the site’s excavation history, we are now able to provide an accurate and near complete profile rendering of not just the cave itself, i.e. the ceiling, rockfall, bedrock morphology, dripline and talus, but also the configuration, thickness, morphology, and distribution of archaeologically defined occupation phases within it. In Fig 15 we are also able to make a calculated interpretation of stratigraphy within the exterior test trench, which has not been appropriately documented or published before.

However, Fig 16C illustrates the risk of interpreting complicated photogrammetric reconstructions without site knowledge. Firstly, the mosaic of section reconstructions spans the full north-south extent of the excavations (Fig 16A and 16B) and so is inevitably distorted by the concave stratigraphy noted in Fig 15. Consequently, Fig 16C is useful as a schematic summary of the site’s stratigraphy, but it is not suitable for detailed analysis of unit thickness and truncation. Secondly from studying the dynamic site wide 3D model, the LSA sediments pinch out towards the east of the site. This appears to result from the low ceiling height relative to the contemporary sediment surfaces within this portion of the cave, an insight which is not apparent in Fig 16C. Here the variable ceiling height represents the maximum height along the north south axis, whereas the LSA was reconstructed using models PGA_31B-C, for which the upper limit of the cave mouth is a better approximation of the locally available space.

**Fig 21:**
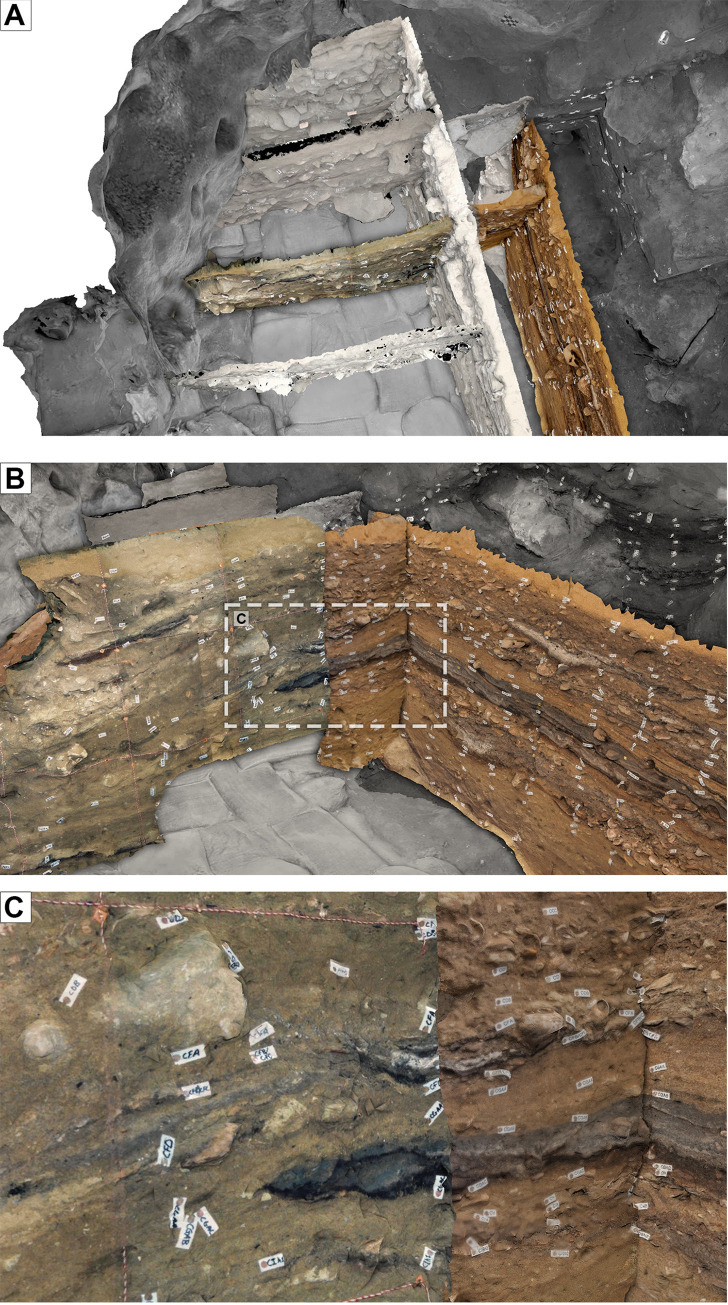
Defining layer extent using retrospective photogrammetry. Combining multiple models from different years (2000 (left) and 2011 (right)) allows spatial reconstruction and visualisation of the true extent of stratigraphic units. Inevitably this form of reconstruction is unable to provide better resolved visual information then the still photographs used to create it. Consequently, the ability to trace stratigraphic layers is at times impeded by a poor quality of the archive images.

The site wide 3D reconstructions of profile walls also enable us to study individual stratigraphic layers, despite constituent quadrants having been uncovered and photographed in different excavation seasons, sometimes a decade or more apart. This is illustrated in Fig 21, which was used to define the extent of a prominent black lens (layer CGAC) at Blombos. For example, while the black lens is readily identifiable on all section wall models (Fig 21), more subtle and thinner sedimentary units become difficult to distinguish where the image quality is lower. This becomes clear when following some of the thinner layers from a 2011 section profile to an earlier 2000 profile.

Consequently, our ability to trace individual stratigraphic units is limited by the quality of the visual information captured (Fig 6). The three most relevant aspects of image quality are in this case, image resolution (Fig 6A and 6B), colour contrast (Fig 6A and 6D) and lighting. In the latter case, strongly directional lighting, striking the surface being photographed at a high angle, creates small shadows which accentuates surface roughness rather than sedimentary characteristics (e.g., compare Fig 6D and 6G).

#### Visual and spatial contextualization of archaeological features, surfaces, and material

Before 3D documentation was implemented at BBC, archaeological features, surfaces, and material were visually interlinked during post-field analyses through orthomosaics or non-georeferenced panoramic images using conventional still photographs. While this approach yielded photorealistic 2D renditions of the archaeological field context (e.g., Fig 17), the spatial accuracy, analytical confidence and ability to build dynamic and geo-referenced datasets were limited. These 2D renditions are also limited to a single perspective and offer only a limited indication of the scale and location of the archaeological features and their relations to the rest of the site (Fig 17B–17E).

By contrast, the retrospective photogrammetric reconstructions of the same areas provide us with a fully georeferenced, 3D rendition of not only the archaeological surface and the artefacts on top, but also their spatial relation to the trench and surrounding area. This digital 3D reconstruction can also be viewed from any angle, and by combining multiple surface models made from photographs taken during multiple seasons, a continuous occupation surface can be reconstructed (which never existed simultaneously in the field). The analytical quality of the 3D model can also be assessed, both through calculating its quantifiable level of confidence (i.e. its spatial accuracy), but also by contextually, spatially and visually evaluating a surface reconstruction of a single quadrant (50x50 cm) in combination with the reconstructed surfaces of adjacent quadrants.

Fig 17 shows an example of how a laterally extensive occupation surface, upon which a scatter of archaeological material was recovered, was excavated, and documented at different times using conventional field photography. The two different sides of the occupation surface (i.e., Fig 17E and 17C) were never physically seen together since their deposition around 80–90 ka [43]. Based on the archival footage, Fig 18 shows a reconstruction of same occupation surface in 3D. In this model, it becomes much more apparent that the archaeological assemblage scattered on the surface is not just spatially associated, but that the different components (ochre stained grind stones, ochre fragments, *Haliotis midae* (South African abalone) shell and bones) are probably behaviourally associated as well.

The combination of these materials have previously been associated with systematic ochre use at the site, and have in some instances been interpreted as ochre processing kits [50]. Whether the reconstructed occupation surface in Fig 18 can be securely linked to a single pigment processing event remains to be tested, but the visual and spatial reconstruction of this surface enable us at least to pose this as a credible hypothesis, and in a much more robust and dynamic way than was possible with any conventional technique.

### Cross-seasonal mapping of recorded archaeological artefacts: Tracking human occupation over time at different analytical resolutions

While the reconstructed 3D models of BBC serve as an excellent starting point for multi-seasonal site and trench exploration, they may also serve as visual and contextual backdrops onto which georeferenced archaeological data can be displayed. This can be done in a basic way, by simply rendering the georeferenced 3D models in combination with georeferenced plotted material (see 3D dots on Fig 19). While the visual effect of these types of combined multi-data 3D models can be useful for getting a general overview of the field context and the general distribution of excavated material, their analytical value is often limited to the dynamic and active exploration conducted by the user within a dedicated 3D viewer.

A more analytically robust and print-friendly approach to visualising and analysing the distribution of 3D recorded archaeological artefacts is offered in Fig 20. Here a 2D orthomosaic section photo from the reconstructed 3D models has been extracted, on top of which the distribution of plotted artefacts is visualised using different techniques. There are benefits and caveats with each approach, but the ability to render quickly plotted point data in different analytical resolutions makes it possible to evaluate which spatial and scalar factors are driving the distribution patterns.

Fig 20A shows a statistical cluster analysis performed on the plotted material recovered, presented in front of the rendered section wall. This type of point-based statistical mapping technique enables us to identify statistically significant point clusters within a global point cloud. The identified clusters are based solely on their spatial relationship to each other (closeness), and irrespective of their archaeological affinity (e.g., archaeological layers). In Fig 20A, where we have indicated archaeological phases using yellow dotted lines, we can observe that some of the generated clusters align well with the archaeological delineation (e.g., cluster 1 is confined to the M3 upper phase). However, it is also true that the distribution of artefacts is not uniform within a single phase either (e.g. clusters 2 and 4, which are located in the eastern and western part of the M1 phase. This latter observation, concerning great lateral variation of the distribution of archaeological material across the cave floor, has been independently reported by Haaland et al. 2021 [39], based on microstratigraphic analysis. As such, the validity of the point cluster analysis appears to be supported and the patterns we see may well be associated with differential use of the cave by its prehistoric inhabitants.

In Fig 20B we have aggregated the data shown in Fig 20A, but with each plotted point assigned to its stratigraphic layer and grid unit (quadrant). To adjust for the fact that the plotted material derives from an excavated 3D volume (50x50 cm quadrant x variable depth), but is visualised only in a 2D profile, we have calculated the quantities of plotted archaeological material per litre recorded sediments per layer per quadrant. This type of 3D-to-2D normalization procedure enables us to map and identify vertical variation in artefact density, following the archaeologically defined stratigraphic units. In Fig 20B we note that the most notable artefact concentration occurs within the M3 upper phase, which again is in accordance with previous observations [39].

In Fig 20C we have aggregated the data shown in Fig 20A, but not assigned the data to any archaeological layer. Consequently, the horizontal spatial resolution of artefact density is better than in Fig 20B as neither limitation of the 50x50 cm quadrants, nor the archaeologically defined layers, factor into the projection. Furthermore, and unlike Fig 20A, the depicted artefact density is less prone to *visual saturation* by high spatial concentrations since the colour scale is defined by the extremes of the dataset. This becomes evident in Fig 20A, where overlapping points do not add to the perception of increased density. In practice, Fig 20A shows that while both the M3 upper phase and the M1 phase represents high-intensity occupation phases, Fig 20C shows that the M3 contains considerably more plotted material than the M1 phase.

### General considerations regarding the application of retrospective photogrammetry to multidecadal excavation archives

#### Cost of retrospective archaeology on long running excavation archive

In order to conduct a photogrammetric reconstruction of an archaeological excavation several resources are required, primary amongst these are raw material, knowledge, equipment and time. The latter requirement is critical to all aspects of this process and a realistic cost-benefit analysis should be conducted before undertaking retrospective photogrammetry of any excavation.

The first factor to consider is the time needed to collect and organise the raw material i.e. the image archive. At Blombos, a significant proportion of the archive consisted of physical slides which were located on a different continent, making it difficult to estimate the time required for this phase of the study at the outset. Digitisation of the slide archive eventually required several hundred hours of work, due to the speed of the scanning equipment, the semi organised storage of the material, and the need to visually review and label resulting digital images.

The second critical resource is knowledge of the site/excavation history and technical expertise in photogrammetry. Acquiring either is time-consuming, but many researchers will already possess one at the outset. Researchers possessing a good understanding of the site and its photographic record will be in a stronger position to determine what potential the available material might have and what questions a photogrammetric reconstruction might help to answer.

The third factor to consider is availability of equipment and/or the cost of acquiring it. Currently both local and cloud based photogrammetric solutions exist, but to explore and interact with the data a certain level of computing power is necessary. In many cases this might already be available, having been acquired for other purposes and the consideration then falls on whether it will be available for the time required. Good slide/photograph scanning equipment is less commonly available. Images could be digitised either by subcontracting the process to a 3^rd^ party or by acquiring appropriate equipment and conducting the process oneself. The former may be costly and gives the researcher less control over decisions made during the digitisation process. The latter is potentially time consuming and repetitive but affords researchers a high degree of quality control and transparency in the process.

In considering the potential rewards obtainable using retrospective photogrammetry, we would stress the importance of having clearly defined aims from the outset–the process is too time-consuming for it to be rational on the basis that one might find “something interesting”. For the same reason, long-term storage and reuse of the reconstructed dataset should also be considered from the outset. It is of critical importance that any digital reconstruction is curated and made available in a manner most likely to become part of future investigations and analysis.

#### What practical challenges were encountered when working with the BBC archive material and how did we solve them?

During the process of digitally reconstructing the BBC excavation we, unsurprisingly, encountered many different technical and practical challenges related to the suboptimal nature of the dataset available. While some issues, such as unwanted objects and people covering parts of a subject, were found across the seasons, the most frequent and difficult problems were encountered when working with the analogue material from the early seasons (1998–2000).

An overview of the most common challenges encountered is presented in Table 4. It should be noted that the varying quality of the analogue archive images and unknown camera parameters caused the quality of 3D models to vary and occasionally precluded the generation of usable 3D models. Furthermore, the manifestation of unsuccessful model generation and workaround solution varied greatly. Therefore, it is likely that future projects will encounter challenges in addition to those listed in Table 4.

**Table 4:** Practical issues encountered and solutions.

Frequently encountered issues	Description	Solution/Work Around
Poor labelling, Unknown year analogue only.	Parts of the analogue image archive was not labelled. The lack of metadata meant that confidently organising the images was impossible, and consequently made it difficult to collect all images that would be relevant for a PG image set.	Visual inspection of all images gradually gave a high familiarity with the image archive and site at various stages. This meant that successfully recognising randomly placed images as related became possible.
Unknown lens parameters/Lack of exif data.	Unlike digital cameras which automatically inscribe each individual image with camera parameters at the time of image capture the analogue images contained no such data. This information is used by the photogrammetric software and the lack of this information consequently negatively impacts it ability to successfully generate correct 3D models.	By gradually including more images over multiple reruns of an image set it became possible to detect individual images which lens parameters seemed to have deviated much from the rest of the images and would cause misalignment/ unsuccessful model generation. These images could then be removed from the image set.
Unwanted artefacts causing a high level of inconsistencies	Some of the images in an image set would contain modern objects such as excavation equipment or people, other times parts of a section would have been changed between some images, either through excavation or section collapse. Models generated with such images will often contain parts of these unwanted elements.	When these images where necessary for the reconstruction we used a masking tool within the photogrammetric solution to manually outline areas with unwanted objects, that could then be excluded from the reconstruction process.
Poor or inconsistent exposure.	Differences in exposure, lighting and white balance in some cases cause the photogrammetric software to miscalculate the surface topography of these areas. The result being warped and untrue model representations.	This was primarily either solved by excluding outlier images or in cases where parts of a problematic image was necessary and usable, we either employed the software’s masking tool or reduced shadows and highlights in a photo editing software (Adobe Photoshop).
Inability to automatically align images due to low overlap and high angles.	Insufficient overlap between images and images captured from an angle that is two different, would prevent the automatic processing by the software solution from aligning images or only aligning some images. This was the case for analogue images captured for the purpose of mosaic compilations and models that had to rely on images captured for vastly different purposes.	One solution was to manually identify and locate features sheared across multiple images and mark them in the software solution. This guided the automatic processing and was found to give good results. A second solution was to locate additional images that had initially been regarded as unsuited due to angle, distance, coverage, or intrusive objects. These could fill in gaps in overlap or provide perspectives form an intermediate angle.
Disagreeable image sets (unknown reasons) Analogue only.	Some image sets that initially looked to be suited for modelling, would prove to be partly or completely unsuccessful and resulted in a variety of warped results.	A solution was to select a few closely related images that provided a good result, and then gradually adding a few more into the dataset through several rounds of processing until one or more disagreeable images were identified and could be removed. Alternatively, an image set might have to be subdivided into two sets and run independently.

This table provides an overview of reoccurring issues encountered when working with the Blombos Cave image archive and solutions and workarounds that were used.

#### Suggestion for best practice workflow

Following our experience with the photographic archive from BBC we suggest the following workflow for best practise (Table 5). This simplified outline is intended as a starting point for projects considering a similar study. However, this must be acknowledged as an idealised strategy, since the varying state and extent of photographic archives across archaeological sites means that there is unlikely to be a universally applicable approach. Consequently, as with most aspects of archaeology, a thorough understanding of the methods used and material available is critical during the planning, execution and interpretation of retrospective photogrammetry work.

**Table 5:** Best practise workflow.

	Task	BBC example
**1**	Identify site specific issues related to inconsistent site recording common in long term excavations.	• 2 coordinate systems (see section titled: Three decades of archaeological excavations: a review of the Blombos Cave documentation systems) • Analogue and digital recording
**2**	Review and determine the nature, state, availability, and quality of the photographic archive.	• Semi consistent organisation of digital archive • Large variety in image quality across all images. • Large analogue image archive with limited descriptive metadata. • Analogue dataset had to be physically collected and digitalized before it could be used.
**3**	Evaluate the analytical potential and limitation of the legacy data.	• Site/record wide analytical comparison and overview potentially facilitated • Frequent black spots due to available legacy data (starting point). • Legacy data has lower resolution.
**4**	Select and process photographs and models relevant for your research questions.	• Section profile reconstruction (see Table 2) • Ochre processing surface reconstruction (see section titled: Reconstructed single context surface)
**5**	Determine the accuracy of 3D models and compare the generated results with the existing (physically known) archaeological record.	• Spatial accuracy assessed through GCP deviation, contemporary physical markers and site grid adherence • Textural fidelity (see section titled: Limitations related to spatial accuracy and textural resolution)
**6**	Determine whether the retrospective 3D models are analytically capable of answering the original research question/agenda considering their resolution and limitations (spatially and visually).	• Site/record-wide overview was created. • Improved understanding of site morphology, stratigraphy and configuration (see section titled: Retrospective photogrammetric reconstruction: a starting point for cross-seasonal and site wide analyses) • Successfully interlinking of material (see section titled: Cross-seasonal mapping of recorded archaeological artefacts: tracking human occupation over time at different analytical resolutions)
**7**	Store and organize 3D models and associated data files in accordance with the FAIR principles of data management.	• Datasets and results structured and prepared for long term storage and reuse.

This table provides a simple step by step suggestion to best practise arranged in a chronological task order, with relevant examples from the project presented in this paper.

## Conclusions

In this paper we have outlined several challenges that are characteristic of many long-term excavations, one of them being changes in field documentation practices over time. The ongoing excavation of the MSA levels at Blombos Cave, South Africa, neatly exemplify these challenges. At this cave site, systematic archaeological documentation has been carried out since 1991, and after three decades of excavations, new excavation data are still being produced. Due to rapid development of visual and spatial recording technology, combined with the implementation of computer-aided digital documentation strategies, the excavation protocol at BBC has changed and been improved numerous times during the project’s lifespan. This has allowed the implementation of more time and cost-effective recording techniques and facilitated the development of more comprehensive, accessible, and interlinked archaeological archives and databases. However, changing excavation protocols have also led to some overarching data integration problems that the present study tries to overcome using archive photos and retrospective photogrammetry.

The archaeological and analytical value of our suggested workflow is exemplified through several case studies from BBC, in which we demonstrate how and why the merging of analogue and digital field documentation can lead to entirely new datasets and results. The main conclusions from our study can be summarized in 5 bullet points:

- *The importance of reviewing data collection history*. Mapping out in detail how the excavation procedures at the site have changed over time, allowed us to formulate targeted and context-specific solutions that both can facilitate, optimize, and evaluate appropriate levels of site-wide data integration.
- *The importance of digitizing archive field photos*. Employing retrospective photogrammetry on BBC, using its rich photo archive, allowed us for the first time to study the excavated portion of the site in its entirety. The resulting dataset allowed the first site-wide assessment of the cave’s overall topography, morphology, and excavated trenches, something that conventional photos or stratigraphy drawings did not permit.
- *The relevance of retrospective photogrammetry for long-running projects*. The workflow suggested in this paper is mostly relevant for long-term projects, where data collection strategies (and field equipment) have changed over time. However, the cost in time and money invested in retrospectively reconstructing a site should be carefully considered relative to the potential reward. In this context, a realistic assessment of the research questions which will be answerable using retrospective photogrammetry needs to be drawn up prior to performing a cost-benefit analysis.
- *The relevance of 3D site exploration for behavioural (anthropological) research in field archaeology*. The datasets produced for BBC demonstrate how retrospective photogrammetry can facilitate robust evaluations of the stratigraphic relationships between major occupational periods and between different occupation phases. The combined 3D datasets also allowed us to map single-context occupation surfaces, that were excavated over multiple seasons, and never seen as a whole. These types of data are invaluable for archaeologists evaluating behavioural aspects of site use, such as occupational intensity and site structure.
- *The importance of reusability of 3D data*. Once a long-term excavation has been reconstructed in 3D through retrospective photogrammetry, it is vital that the data are made available for everyone working on the site and for future researchers. While the use of this data will require a basic level of competence with photogrammetry and GIS software, these skills form a standard part of modern archaeological curricula. Therefore, photogrammetric data, and their associated metadata, must be available and readily understandable for future researchers. As a minimum, this requires that the material is curated in a manner which allows seamless integration of newly recorded data produced as part of ongoing excavations using the current recording strategy.

## Supporting information

S1 File 3D model of Blombos Cave as it existed in March 2020. This model combines photogrammetric recordings from 2013 and 2019. It has been simplified to reduce demands on computer hardware.
(PDF)

S2 File 3D model of Blombos Cave containing a selection of reconstructed section profiles. This model combines the most recent total site model of Blombos Cave (which integrates data from 2013 and 2019) with a selection of reconstructed section profiles created using images from the image archive. The model has been simplified to reduce demands on computer hardware.
(PDF)
