# Supplementary material for: Reconcilable differences: Using retrospective photogrammetry to bridge the divide between analogue and digital site data collected during long-term excavation projects
Source: PLoS One. 2024 Nov 21;19(11):e0310741. doi: 10.1371/journal.pone.0310741 (PMC11581232; doi:10.1371/journal.pone.0310741)
Supplement: S1 File — This model combines photogrammetric recordings from 2013 and 2019. It has been simplified to reduce demands on computer hardware. (PDF) [file pone.0310741.s001.pdf]

**S1 Model. 3D model of Blombos Cave as it existed in March 2020.** This model combines photogrammetric recordings from 2013 and 2019. It has been simplified to reduce demands on computer hardware.
