# Supplementary material for: Reconcilable differences: Using retrospective photogrammetry to bridge the divide between analogue and digital site data collected during long-term excavation projects
Source: PLoS One. 2024 Nov 21;19(11):e0310741. doi: 10.1371/journal.pone.0310741 (PMC11581232; doi:10.1371/journal.pone.0310741)
Supplement: S2 File — This model combines the most recent total site model of Blombos Cave (which integrates data from 2013 and 2019) with a selection of reconstructed section profiles created using images from the image archive. The model has been simplified to reduce demands on computer hardware. (PDF) [file pone.0310741.s002.pdf]

S2 Model. 3D model of Blombos Cave containing a selection of reconstructed section profiles. This model combines the most recent total site model of Blombos Cave (which integrates data from 2013 and 2019) with a selection of reconstructed section profiles created using images from the image archive. The model has been simplified to reduce demands on computer hardware.
